# Supplementary material for: HnRNP-L-regulated circCSPP1/miR-520h/EGR1 axis modulates autophagy and promotes progression in prostate cancer
Source: Mol Ther Nucleic Acids. 2021 Oct 19;26:927–44. doi: 10.1016/j.omtn.2021.10.006 (PMC8560719; doi:10.1016/j.omtn.2021.10.006)
Supplement: Document S1. Tables S1–S3 and Figures S1–S7 [file mmc1.pdf]

OMTN, Volume *26*

Supplemental information

HnRNP-L-regulated circCSPP1/miR-520h/*EGR1* axis modulates autophagy and promotes progression in prostate cancer

Jianming Lu, Chuanfan Zhong, Junqi Luo, Fangpeng Shu, Daojun Lv, Zezhen Liu, Xiao Tan, Shuo Wang, Kaihui Wu, Taowei Yang, Weibo Zhong, Bin Wang, Yanfei Chen, Yuehan Li, Zhenyu Jia, Yaguang Zou, Weide Zhong, and Xiangming Mao

Supplementary Table 1 Sequence of primers, small RNAs and probes used in the study.

Primers		
Gene	**Forward (5'-3')**	**Reverse (5'-3')**
HnRNP-L	TTGTGGCCCTGTCCAGAGAATT	GTTTGTGTAGTCCCAAGTATCCTG
convergent-circCSPP1	TGTGAAAATTCAGAGGGTCCTA	TTGGAAATGCCCACTTCTTC
divergent-circCSPP1	TGAAGATTTGCGCAGTGGAC	GAGCATCCCTGCAAAAGGAC
convergent-GAPDH	GTCAGTGGTGGACCTGACCT	TGACAAAGTGGTCGTTGAGG
divergent-GAPDH	CACACTGAATCTCCCCTCCT	GTCCACCACCCTGTTGCT
CSPP1	TGAAGATTTGCGCAGTGGAC	GAGCATCCCTGCAAAAGGAC
GAPDH	ACAGTCAGCCGCATCTTCTT	GACAAGCTTCCCGTTCTCAG
EGR1	CACCTGACCGCAGAGTCTTTT	GGCCAGTATAGGTGATGGGG
β-actin	AGCGAGCATCCCCCAAAGTT	GGGCACGAAGGCTCATCATT
S1-pre-circCSPP1	TGGGCGACAGAGTGA	GAGTGGGACTTGGAAAC
S2-pre-circCSPP1	TCCCTCCCTTTCTTCT	ACAACTCAGAATAGAACCC
S3-pre-circCSPP1	TGTAGAGGAGCACTATTTCC	GAGTGGGACTTGGAAAC
S4-pre-circCSPP1	GTAGCTCTTTGCCTTCT	GTACAGGAGAAGGGACAT
S5-pre-circCSPP1	GTGCGGCATTACCAG	TGATCGCCATTCTAACT

siRNA/miRNA mimics/ inhibitors Sequence		
	**sense 5'-3'**	**antisense 5'-3'**
si-HnRNP-L	GCUUGGAUCAAUCUAAGAUTT	AUCUUAGAUUGAUCCAAGCTT
circCSPP1-sh1	AUGGUGUCUCCCAGUGCUCTT	GAGCACUGGGACACACCAUTT
circCSPP1-sh2	GUCUCCCAGUGCUCCAGACTT	GUCUGGAGCACUGGGAGACTT
hsa_miR-520h mimics	ACAAAGUGCUUCCCUUUAGAGU	UCUAAAGGGAAGCACUUUGUUU
hsa_miR-520h inhibitors	ACUCUAAAGGGAAGCACUUUGU	

FISH probes targeting circCSPP1/miR-520h/*EGR1*		
	**Sequence 5'-3'**	**modification**
circCSPP1	TGTTTCATTGTCTGGAGCACTGGGAGACACCATTTCACCA	CY3/FAM
hsa_miR-520h	ACTCTAAAGGGAAGCACTTTGT	CY3
EGR1	AAAAGCGGCCAGTATAGGTGAT	FAM

Pulldown probes targeting circCSPP1/miR-520h		
	**Sequence 5'-3'**	**modification**
circCSPP1	UUCAUUGUCUGGAGCACUGGGAGACACCAUUUCACC	5’ biotin
Oligo probes	UUCUCCGAACGUGUCACGUTT	5’ biotin
hsa_miR-520h_sense	ACAAAGUGCUUCCCUUUAGAGU	3’ biotin
hsa_miR-520h_antisense	ACUCUAAAGGGAAGCACUUUGU	-

Supplementary Table 2 Clinical characteristics of the prostate cancer patients.

Parameter			
Age at diagnosis (mean ± SD)		72 ± 3.53	
Clinical stage, n(%)	T1	1	10%
	T2	6	60%
	T3	3	30%
Gleason score, n(%)	≤6	3	30%
	=7	3	30%
	≥8	4	40%
PSA at diagnosis (ng/ml), n(%)	0-3.9	1	10%
	4-9.9	3	30%
	≥10	6	60%

Supplementary Table 3 Clinical characteristics of the benign prostate hyperplasia.

Parameter			
Age at diagnosis (mean ± SD)		70.7 ± 3.92	
Pathological diagnosis, n (%)	Benign	10	100%
	Cancer	0	0%
PSA at diagnosis (ng/ml), n (%)	0-3.9	10	100%
	4-9.9	0	0%
	≥10	0	0%

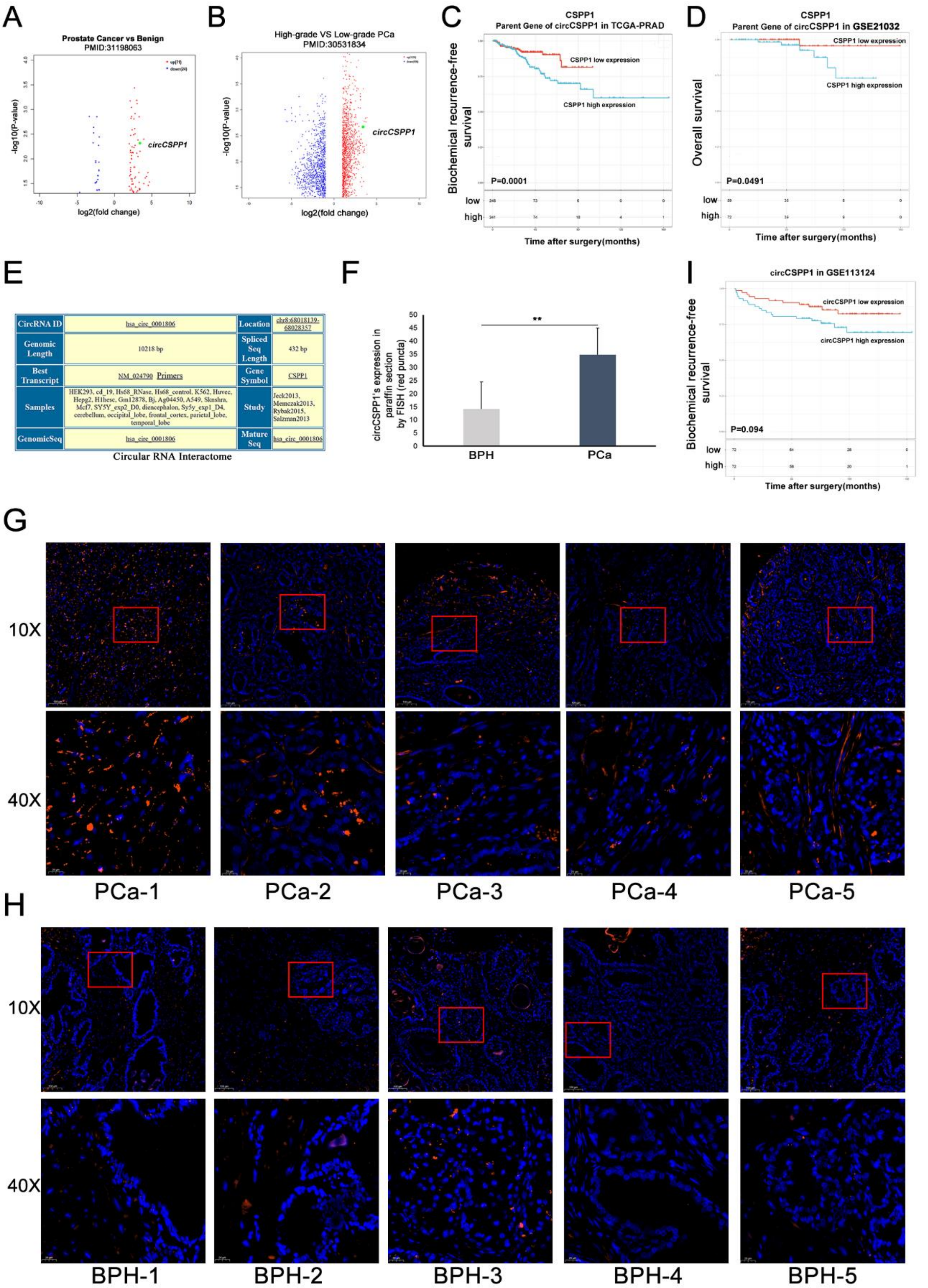

Supplementary Figure 1 circCSPP1 is upregulated in PCa. (A) The scatter diagram shows the most differentially expressed circRNAs in PCa tissues and benign tissues. (B) Dysregulated circRNAs expression were shown in 5 pairs of High-grade and low-grade PCa samples. (C) Biochemical recurrence-free survival time about parent gene of circCSPP1 analysis in TCGA database (D) Overall survival (OS) time data for prostate

cancer patients from the Kaplan-Meier Plotter analysis. (E) Annotation of circCSPP1 from Circular RNA Interactome. (F) Expression of circCSPP1 in clinical samples. (G) circCSPP1 is detected by FISH in PCa paraffin section (DAPI, blue puncta; circCSPP1, red puncta). (H) circCSPP1 is detected by FISH in BPH paraffin section (DAPI, blue puncta; circCSPP1, red puncta). (I) KM plot indicated the high circCSPP1 expression is correlated with faster BCR in patients with PCa though the log-rank p value = 0.094. Data are shown as means ± SD, Student's t test, two-tailed, **P<0.01.

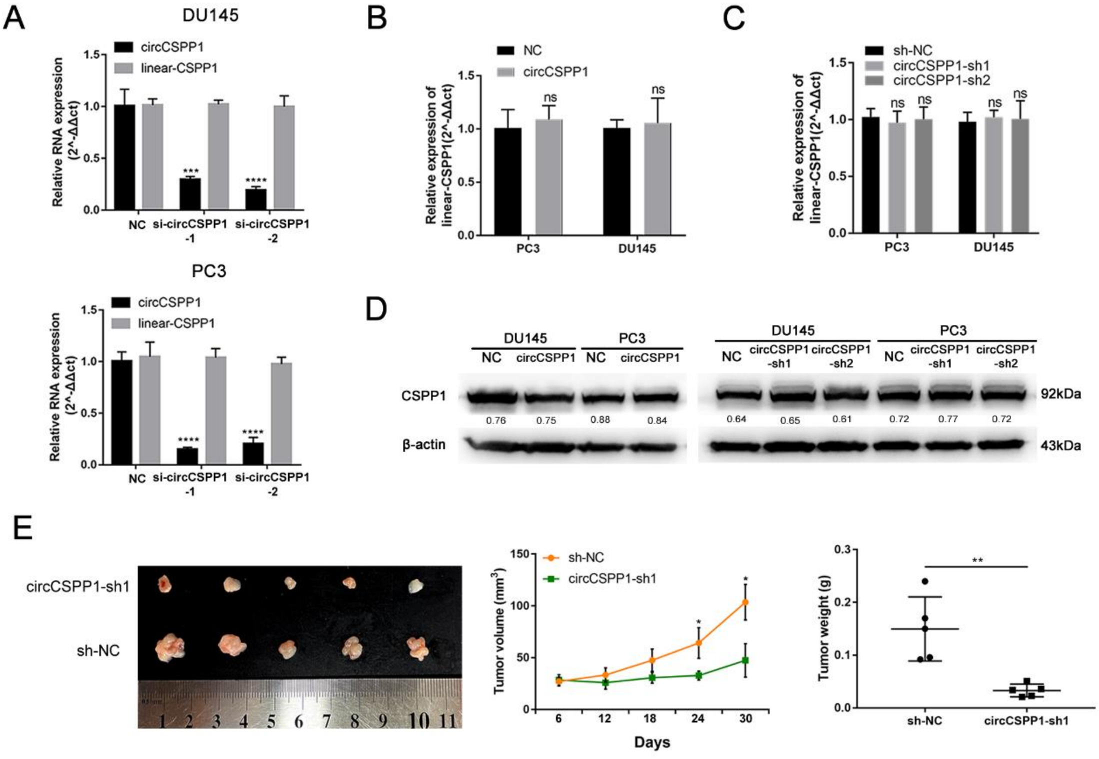

Supplementary Figure 2 circCSPP1 modulates PCa proliferation positively *in vivo* without interfering the expression of linear-*CSPP1*. (A) qRT-PCR validation for the transfection efficiency of siRNA targeting circCSPP1 and its influence on the expression of linear-CSPP1. (B, C) qRT-PCR validation for the transfection efficiency of lentivirus expressing circCSPP1 and sh-circCSPP1 and their impacts on the expression of linear-*CSPP1*. (D) Overexpression or knockdown of circCSPP1 barely affected the protein expression of CSPP1. (E) Image of subcutaneous tumors derived from PC3 cell transfected with vector or circCSPP1-sh1 in the xenograft model. Tumor volumes were measured every 6 days up to 30 days and the final tumor weight was calculated. Data are shown as means ± SD, Student's t test, two-tailed, ****P<0.0001, ***P<0.001, **P<0.01,*P<0.05, ns, not significant.

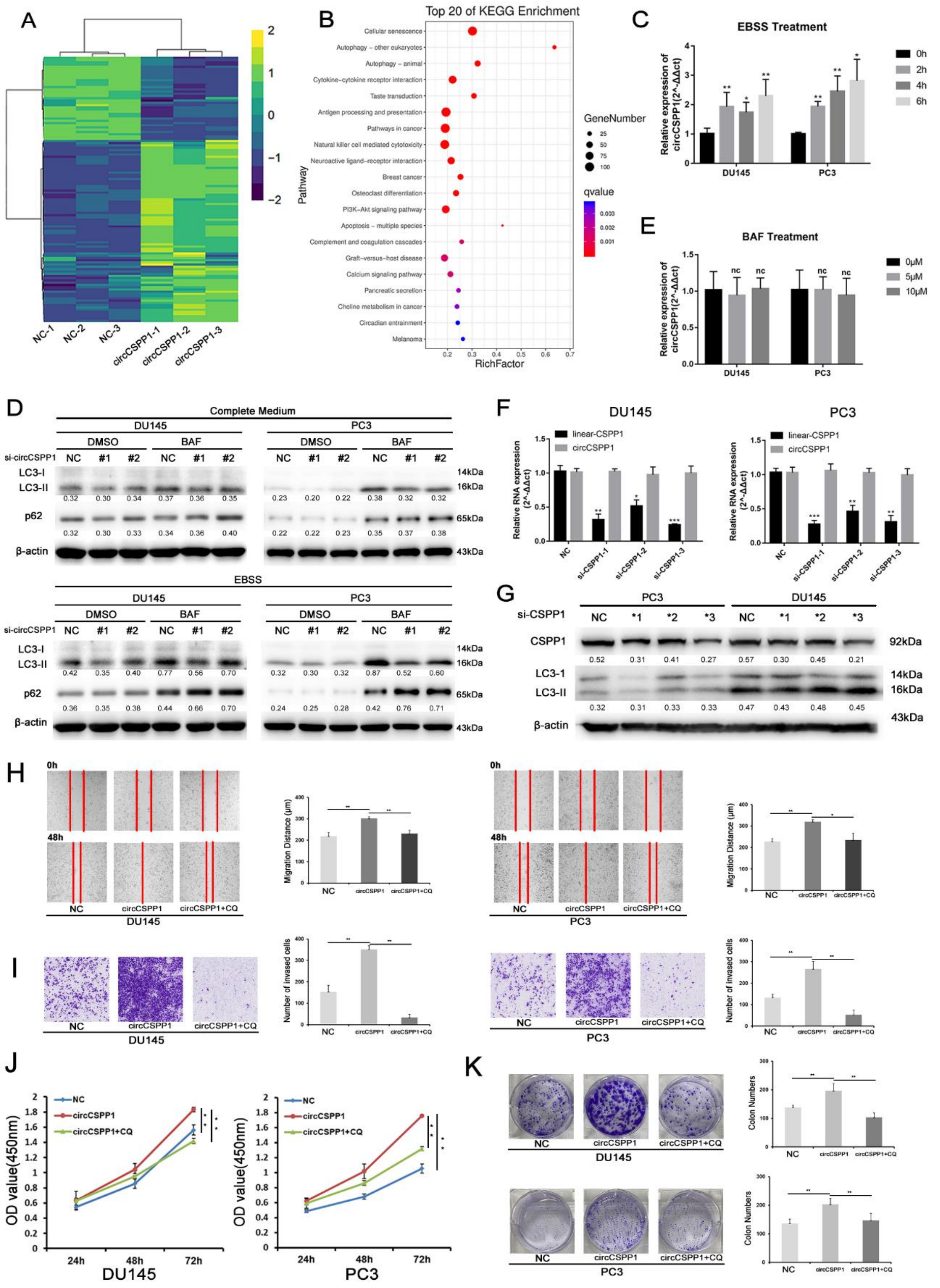

Supplementary Figure 3 circCSPP1 promotes PCa progression via activating autophagy. (A) Heatmap representing unsupervised hierarchical clustering of genes regulated by circCSPP1 based on next-generation sequencing (GSE158975) analysis. (B) KEEG analysis displays the enrichment pathways in circCSPP1 overexpressed cell lines.

(C) Increments of circCSPP1 over time by autophagy induction with EBSS. (D) Western blotting detected the conversion of LC3-II and P62 in complete medium or EBSS condition under DMSO/BAF treatment after si-circCSPP1 transfection. (E) Expression of circCSPP1 remains unchanged under treatment with BAF in different concentration. (F) qRT-PCR validation for the transfection efficiency of siRNA targeting linear-*CSPP1* and its impact on the expression of circCSPP1. (G) Knockdown of linear-*CSPP1* significantly decreased the protein level of CSPP1 but exhibited no effect on LC3-II and P62 expression. (H, I) The migration and invasion capabilities of DU145 and PC3 transfected with circCSPP1 were determined by the wound healing and transwell assays with or without CQ. (J, K) The proliferative ability of DU145 and PC3 transfected with circCSPP1 was determined through the colony formation and CCK-8 assays with or without CQ. Data are shown as means ± SD, Student's t test, two-tailed, ***$P<0.001$, **$P<0.01$, *$P<0.05$, ns, not significant.

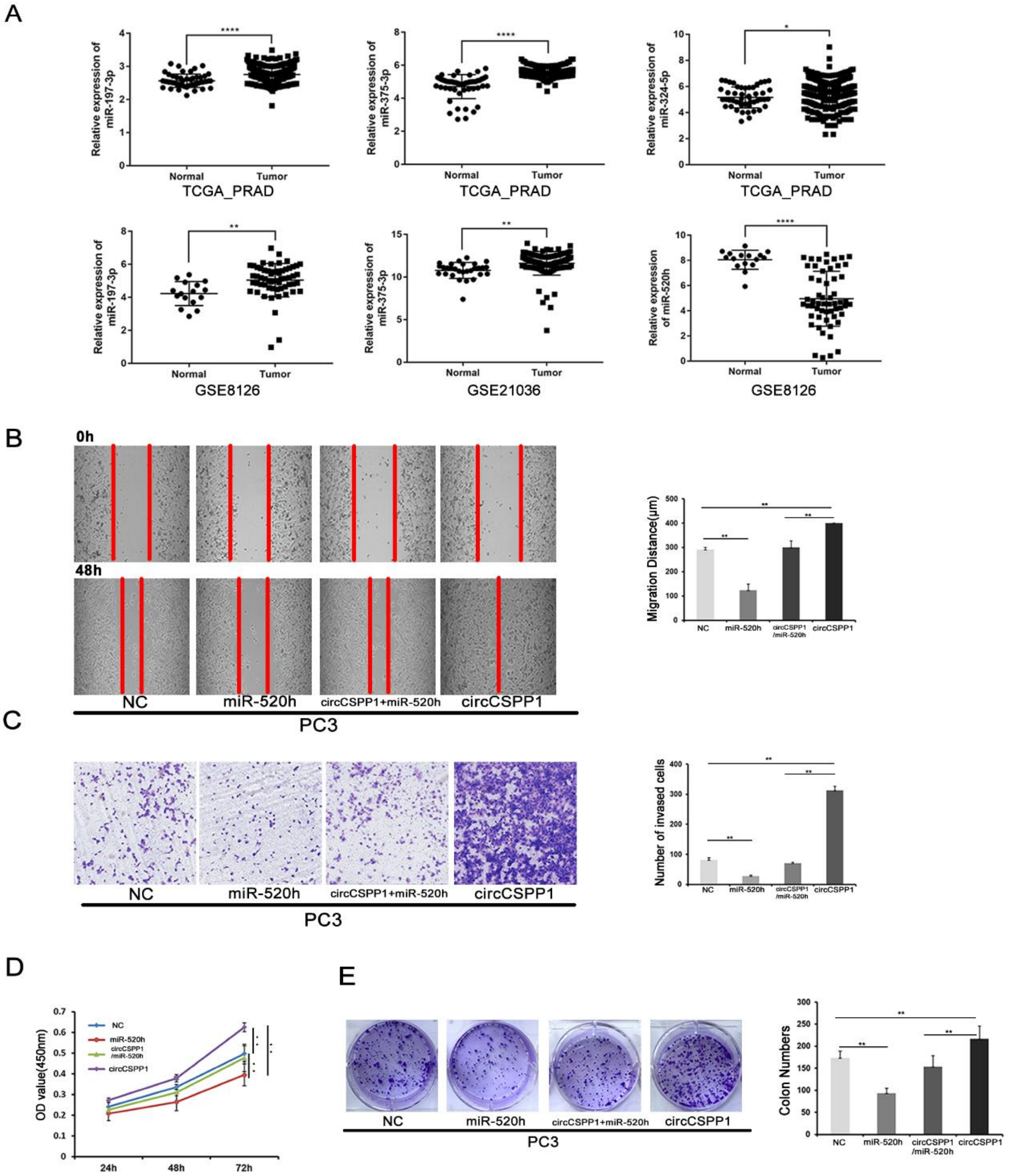

Supplementary Figure 4 circCSPP1 promotes PCa progression through sponging off miR-520h. (A) Differentially expression of putative miRNAs in TCGA and GEO databases, and miR-520h was downregulated in PCa while miR-432-5p was unknown. (B, C) The migration and invasion capabilities of DU145 and PC3 transfected with circCSPP1 and/or miR-520h mimics were determined with the wound healing and transwell assays. (D, E) The proliferative ability of DU145 and PC3 transfected with circCSPP1 and/or miR-520h mimics was determined through the colony formation and CCK-8 assays. Data are shown as means ± SD, Student's t test, two-tailed, ****P<0.0001, **P<0.01, *P<0.05, ns, not significant.

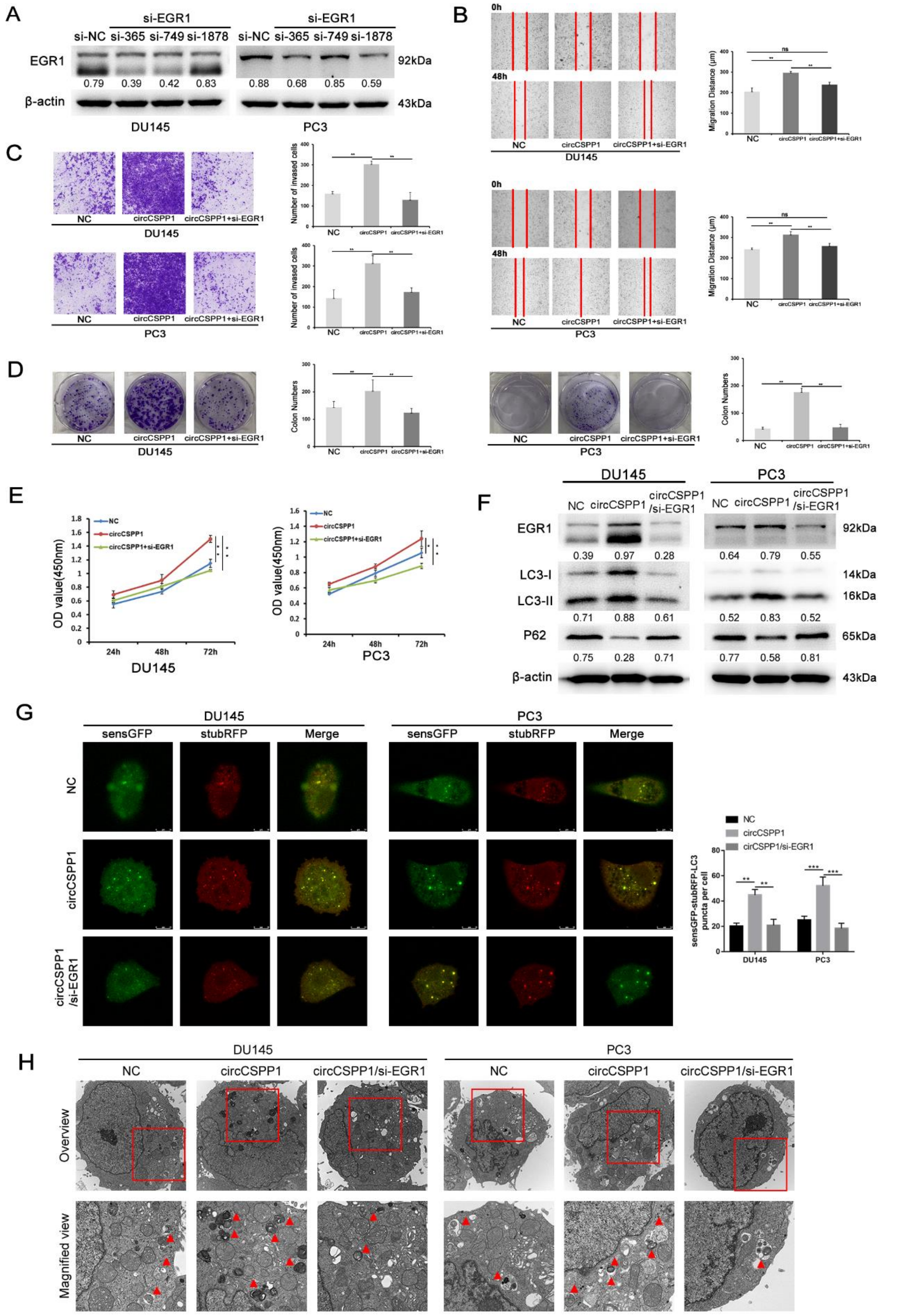

Supplementary Figure 5 Knockdown of *EGR1* attenuated circCSPP1 induced progression and autophagy in PCa *in vitro*. (A) Western blot validation for the

transfection efficiency of siRNA targeting *EGR1*. (B, C) The migration and invasion capabilities of DU145 and PC3 transfected with vector or circCSPP1, or co-transfected with circCSPP1 and si-*EGR1* were determined with the wound healing and transwell assays. (D, E) The proliferative ability of DU145 and PC3 transfected with vector or circCSPP1, or co-transfected with circCSPP1 and si-*EGR1* was determined through the colony formation and CCK-8 assays. (F) Western blotting verified knockdown of *EGR1* attenuated the circCSPP1 induced increment of the conversion of LC3-I/LC3-II and P62 degradation. (C) The accumulation of LC3-II puncta (yellow, green and red overlap) was detected in DU145 and PC3 after transfection of circCSPP1 or co-transfection of circCSPP1 and si-*EGR1*. (D) Autophagosomes (arrow) were observed by transmission electron microscopy (TEM) in DU145 and PC3 after transfection of circCSPP1 or co-transfection of circCSPP1 and si-*EGR1*. Data are shown as means ± SD, Student's t test, two-tailed, ***P<0.001, **P<0.01, *P<0.05, ns, not significant.

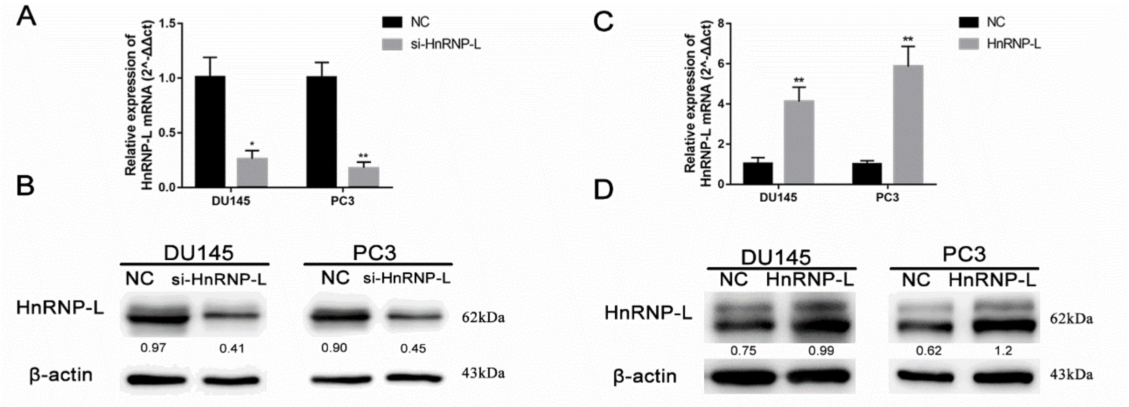

Supplementary Figure 6 Transfection efficiency of si-HnRNP-L and HnRNP-L overexpression in DU145 and PC3 cell lines. (A, B) qRT-PCR and Western blot validation for the transfection efficiency of siRNA targeting HnRNP-L. (C, D) qRT-PCR and Western blot validation for the transfection efficiency of lentivirus expressing HnRNP-L. Data are shown as means ± SD, Student's t test, two-tailed, $**P<0.01$, $*P<0.05$.

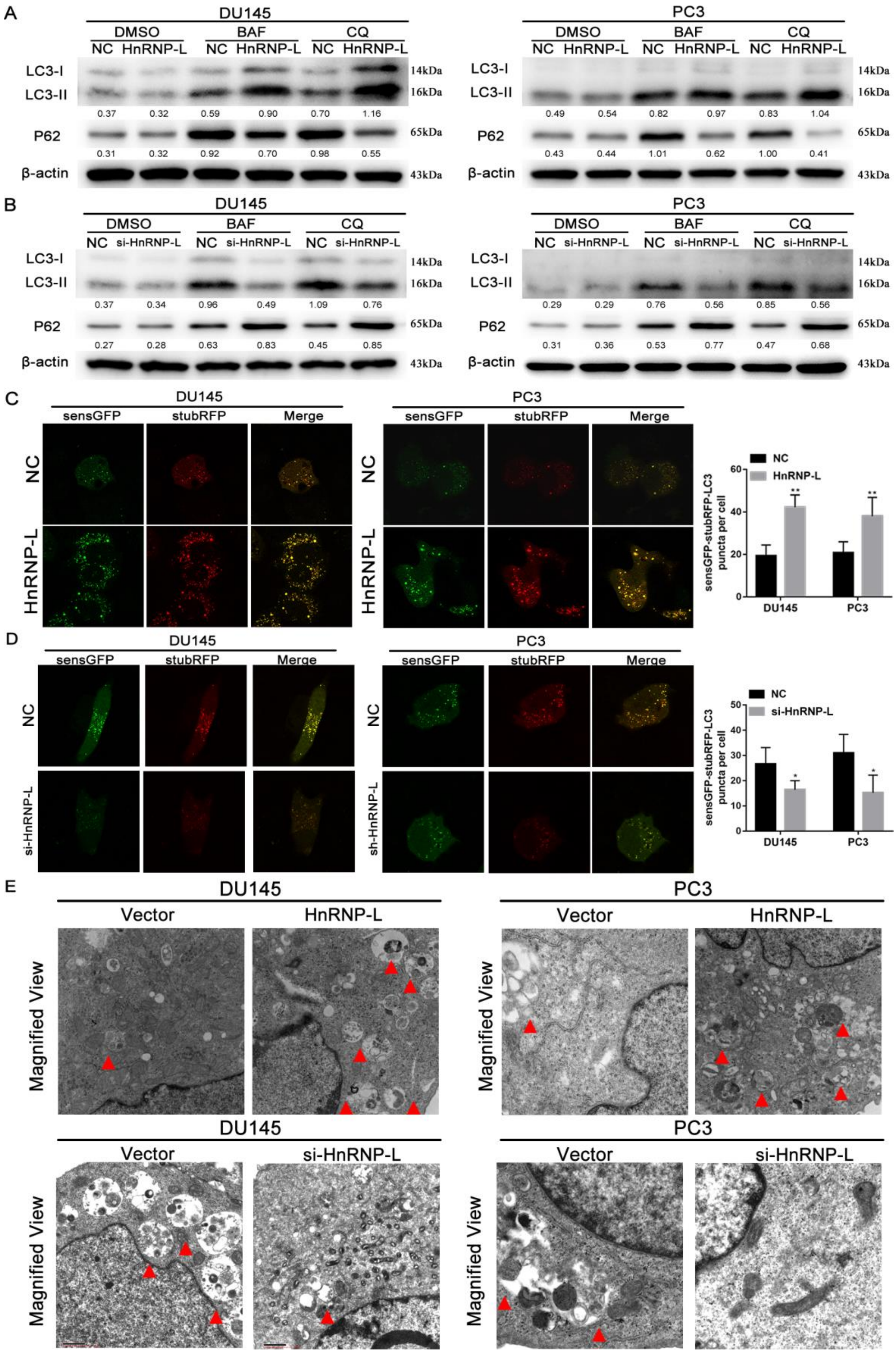

Supplementary Figure 7 Overexpression or knockdown of HnRNP-L augmented or impeded autophagy in DU145 and PC3 cell lines. (A, B) Western blotting verified

overexpression or knockdown of HnRNP-L promoted or inhibited the conversion of LC3-I/LC3-II and P62 degradation. (C) The accumulation of LC3-II puncta (yellow, green and red overlap) was detected in DU145 and PC3 after overexpression or knockdown of HnRNP-L. (D) Autophagosomes (arrow) were observed by transmission electron microscopy (TEM) in DU145 and PC3 after overexpression or knockdown of HnRNP-L. Data are shown as means ± SD, Student's t test, two-tailed, **$P<0.01$, *$P<0.05$.
