# Supplementary material for: HnRNP-L-regulated circCSPP1/miR-520h/EGR1 axis modulates autophagy and promotes progression in prostate cancer
Source: Mol Ther Nucleic Acids. 2021 Oct 19;26:927–44. doi: 10.1016/j.omtn.2021.10.006 (PMC8560719; doi:10.1016/j.omtn.2021.10.006)
Supplement: Document S2. Article plus supplemental information [file mmc2.pdf]

Molecular Therapy
Nucleic Acids
Original Article

HnRNP-L-regulated circCSPP1/miR-520h/*EGR1* axis modulates autophagy and promotes progression in prostate cancer

Jianming Lu,[1,9] Chuanfan Zhong,[1,9] Junqi Luo,[1,9] Fangpeng Shu,[1] Daojun Lv,[2] Zezhen Liu,[2] Xiao Tan,[3] Shuo Wang,[1] Kaihui Wu,[1] Taowei Yang,[1] Weibo Zhong,[1] Bin Wang,[4] Yanfei Chen,[4] Yuehan Li,[5] Zhenyu Jia,[6] Yaguang Zou,[7] Weide Zhong,[8] and Xiangming Mao[1]

[1]Department of Urology, Zhujiang Hospital, Southern Medical University, Guangzhou 510282, Guangdong, P.R. China; [2]Department of Urology, Minimally Invasive Surgery Center, the First Affiliated Hospital of Guangzhou Medical University, Guangdong Key Laboratory of Urology, Guangzhou Institute of Urology, Guangzhou 510120, Guangdong, P.R. China; [3]Department of Urology, The Affiliated Hospital of Southwest Medical University, Luzhou 646000, Sichuan, P.R. China; [4]Department of Urology, Affiliated Cancer Hospital & Institute of Guangzhou Medical University, 78 Hengzhigang Road, Guangzhou, Guangdong 510095, P.R. China; [5]College of Letters, Arts, and Sciences, University of Southern California, Los Angeles, CA 90089, USA; [6]Department of Botany and Plant Sciences, University of California, Riverside, CA 92521, USA; [7]Department of Stomatology, Nanfang Hospital, Southern Medical University, Guangzhou 510515, Guangdong, P.R. China; [8]Guangdong Provincial Institute of Nephrology, Nanfang Hospital, Southern Medical University, Guangzhou 510515, P.R. China

The circRNAs, a new subclass of non-coding RNAs that are catalyzed by RNA-binding proteins (RBPs), have been reported to be associated with the progression of multiple types of cancer. We previously discovered that heterogeneous nuclear ribonucleoprotein L (HnRNP-L), a multi-functional RBP, is associated with pro-proliferation and anti-apoptosis activities in prostate tumor cells. In this study, we aim to establish the biological relevance of circCSPP1 (a newly discovered signature circRNA in prostate cancer [PCa]) and HnRNP-L to prostate cancer progression. First, we demonstrated that circCSPP1 expression was higher in prostate cancer tissues than in benign tissues and higher in prostate cancer cells than in benign cells. Then, the *in vitro* gain- and loss-of-function experiments showed that the circCSPP1 expression in prostate cancer cells was regulated by HnRNP-L, and the increased circCSPP1 significantly induced autophagy, which led to an enhanced potential in proliferation, migration, and invasion of prostate cancer cells. These results were consistent with the *in vivo* experiment where increased or decreased circCSPP1 was associated with higher or slower growth rate in grafted tumors. Finally, we demonstrated the potential competing endogenous RNA network, involving circCSPP1, miR-520h, and early growth response factor 1 (*EGR1*), in prostate cancer cells, which may play an important role in prostate cancer progression. Our study indicated that the increase in circCSPP1 in prostate cancer, which may be catalyzed by HnRNP-L, can induce cellular autophagy through the circCSPP1-miR-520h-*EGR1* axis, leading to the progression of prostate tumor. This newly discovered circRNA biomarker may be used for clinical prognosis of prostate cancer as well as for development of novel therapy plans.

INTRODUCTION

Prostate cancer remains the second leading cause of cancer death in American men, only behind lung cancer.[1] The incidence and mortality of prostate cancer (PCa) are both rising steadily in multiple countries.[2] Many molecular mechanisms have been proposed for formation and progression of prostate cancer, including DNA somatic mutations, harmful gene fusions, irregular methylation, and aberrant RNA splicing.[3,4] Moreover, a recent study showed that knockout of *ATG7* (a key autophagic regulator) inhibited prostate cancer progression in castrate-resistant or castrate-sensitive prostate cancer, suggesting that dysfunctional autophagy may also be associated with prostate cancer progression.[5] However, the current knowledge of the disease is so limited that it barely accounts for the heterogeneous nature of prostate tumors. New insights at molecular levels and organelle levels are needed to improve our understanding of this complex disease and to develop novel tools for diagnosis and prognosis and new strategies for personalized treatment.[6]

Received 17 January 2021; accepted 5 October 2021;
https://doi.org/10.1016/j.omtn.2021.10.006.

[9]These authors contributed equally

Correspondence: Xiangming Mao, Department of Urology, Zhujiang Hospital, Southern Medical University, Guangzhou 510282, Guangdong, P.R. China.
E-mail: mxm631221@126.com

Correspondence: Weide Zhong, Guangdong Provincial Institute of Nephrology, Nanfang Hospital, Southern Medical University, Guangzhou 510515, P.R. China.E-mail:
E-mail: zhongwd2009@live.cn

Correspondence: Yaguang Zou, Department of Stomatology, Nanfang Hospital, Southern Medical University, Guangzhou 510515, Guangdong, P.R. China.
E-mail: 18620050609@163.com

This is an open access article under the CC BY license (http://creativecommons.org/licenses/by/4.0/).

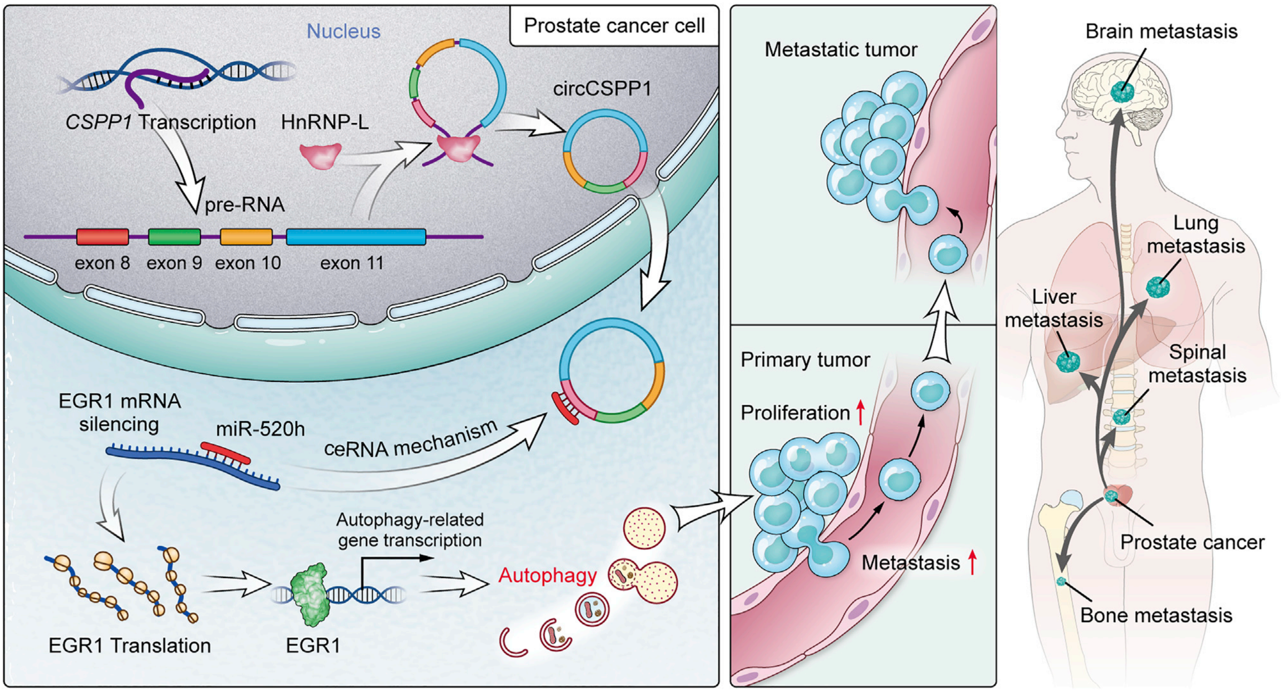

Figure 1. A schematic model presenting that circCSPP1 is upregulated by HnRNP-L and the circCSPP1/miR-520h/EGR1 axis promotes prostate cancer progression through autophagy

The majority of the human genome consists of non-coding DNAs, many of which are transcribed to non-coding RNAs, including micro-RNAs (miRNAs) and long non-coding RNAs (lncRNAs).[7] Research has shown that non-coding RNAs may play an important role in the progression of prostate cancer.[8] Recently, mounting attention has been brought to circRNAs, a newly uncovered type of non-coding RNAs, for their potential effect on tumorigenesis and progression.[9] Catalyzed by certain RNA-binding proteins (RBPs), precursor RNAs are spliced to form single-stranded loops—a covalently closed structure.[10] Thus, this kind of RNA is relatively more stable than others in body fluid, such as blood plasma, urine, and exosomes, promising to be an ideal easy-to-detect biomarker.[11] RNA splicing, a critical process in genomic transcription, may get involved in initiation and exacerbation of tumors via its ability to regulate genomic stability and transcriptome and chromatin organization.[12,13] For example, the alternative RNA splicing of androgen-receptor (*AR*) splice variant 7 messenger RNA (AR-V7) may cause the resistance of *AR* pathway inhibitors, leading to the progression of prostate cancer.[14] When it comes to circRNAs, back-splicing is mainly responsible for circRNA biogenesis, which is different from alternative splicing in linear RNAs.[10] It has been hypothesized that the upstream site and downstream site of the circRNA carry the same repeat elements, and the participating RBPs bring these two sites into proximity to constitute a loop structure.[15,16] We have discovered that heterogeneous nuclear ribonucleoprotein L (HnRNP-L), a multi-functional RBP, is associated with pro-proliferation and anti-apoptosis activities in prostate tumor cells.[17,18] Our study indicated that increased expression of HnRNP-L in prostate cancer cells accelerates disease progression, but the actual function of HnRNP-L in prostate cancer remains opaque. Given its involvement in the formation of circRNAs, this unique RBP is highly likely to influence prostate tumors through regulating key circRNAs to promote cancer development. For example, our analysis based on four different publicly available datasets showed that circCSPP1 has higher expression in prostate cancer tissue than in benign tissue, or higher expression in high-grade prostate cancer tissue than in low-grade prostate cancer tissue, while an RNA immunoprecipitation (RIP) assay and mini-gene system data demonstrated that circCSPP1 was modulated by HnRNP-L (see Results).

In the current study, we aimed to establish the carcinomatous relevance of interactivity between HnRNP-L and circCSPP1 in prostate tumor. Bioinformatics analysis indicated that circCSPP1 can interfere with the complementary binding between miR-520h and early growth response factor 1 (*EGR1*), a well-known oncogene that causes tumorigenesis and metastasis in prostate cancer through the regulation of cell autophagy.[19] Therefore, we hypothesized that HnRNP-L regulates the circCSPP1-miR-520h-*EGR1* axis to promote autophagy in prostate cancer cells, leading to tumor proliferation and metastasis. As indicated in Figure 1, the increased expression of HnRNP-L likely upregulates the expression of circCSPP1; within tumor cells, circCSPP1 may sponge off miR-520h and thereafter unleash *EGR1*. As a result, the highly expressed EGR1 protein will upregulate autophagy-related gene expression at the transcriptional level, promoting

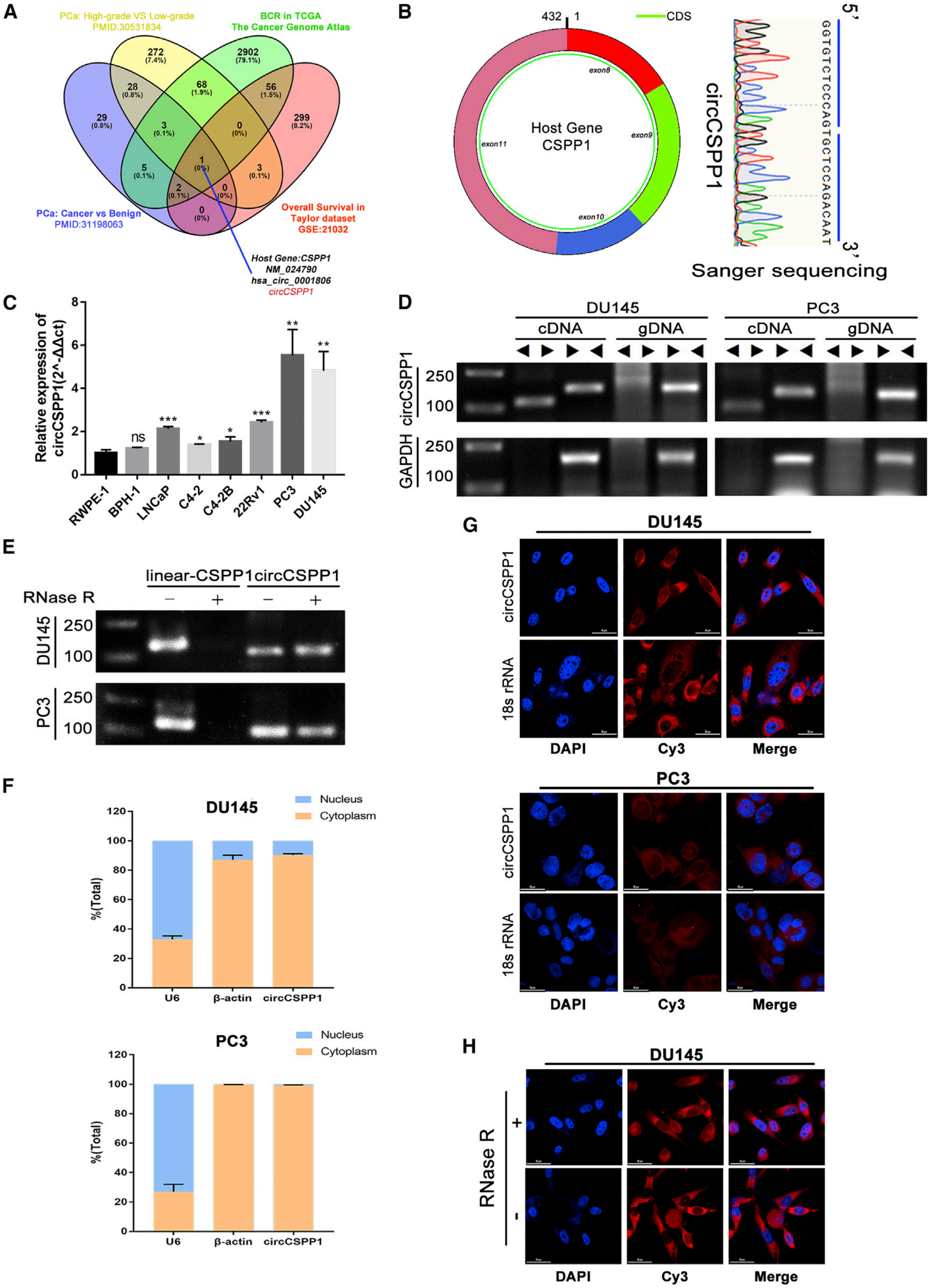

Figure 2. Analysis of circCSPP1 characteristics in human prostate cancer tissues and cell lines

(A) Venn diagram shows circCSPP1 is the only gene in the overlap. (B) Sanger sequencing detected the back-splicing site of circCSPP1. (C) Naive expression of circCSPP1 in prostatic cell lines. Student's t test, ****p < 0.0001, **p < 0.01, *p < 0.05. (D) RNase R resistance test of circCSPP1. (E) Divergent and convergent primers were used to detect

(legend continued on next page)

tumor proliferation and metastasis. In the study, we carried out various experiments to validate these hypotheses, including western blot, confocal microscopy, and transmission electron microscopy (TEM) for gauging autophagy flux, loss-of-function and gain-of-function assays *in vitro* and *in vivo*, RIP and mini-gene systems, and a series of bioinformatics analyses. Our data provided insights into a circRNA-involved regulatory network that induces autophagy in prostate cancer cells, which promotes tumor progression.

RESULTS

circCSPP1 is upregulated in prostate cancer

Four publicly available datasets, including two microarrays,[20,21] GSE21036 and TCGA_PARD, were analyzed, indicating that circCSPP1 was always associated with prostate cancer and its progression (Figure 2A). The results showed that the expression of circCSPP1 was higher in prostate cancer tissues than that in benign tissues, and it was also upregulated in high-grade prostate cancer tissues, compared to the low-grade prostate cancer tissues (Figures S1A, S1B, S1G, and S1H). The survival analysis indicated that the mRNA expression level of CSPP1, the parent gene of circCSPP1, was positively associated with the biochemical recurrence (BCR), free survival, and overall survival (OS) in prostate cancer (i.e., patients with higher *CSPP1* expression had significantly worse outcomes than those with lower *CSPP1* expression) (Figures S1C and S1D). We found that circCSPP1, whose annotation is shown in the Circular RNA Interactome (Figure S1E), is derived from exons 8–11, and the back-splicing site was identified by Sanger sequencing (Figure 2B). To further verify the differential expression of circCSPP1 in prostate cancer cells (C4-2, C4-2B, LNCaP, 22Rv1, DU145, PC3) or benign cells (RWPE-1, BPH-1) as well as prostatic tissues, we designed qPCR primers and fluorescent probes specifically targeting the back-splicing site of circCSPP1. The results of qPCR showed that the expression of circCSPP1 was significantly higher in prostate cancer cells than in benign cells, and it was also higher in prostate cancer tissues than in non-cancerous tissues (Figure 2C; Figures S1F–S1H). The experiment of RNase R treatment showed that circCSPP1 was resistant to the RNase R digestion activity and remained stable (Figure 2D). When compared with the genomic DNA (gDNA) group, circCSPP1 could be amplified by divergent primers in cDNA samples, and their PCR products were validated by agarose gel electrophoresis (Figure 2E). Next, subcellular fractionation and fluorescence *in situ* hybridization (FISH) analysis showed that circCSPP1 was mainly localized in the cytoplasm in DU145 and PC3 (Figures 2F and 2G) and sustained stability under the treatment of RNase R digestion (Figure 2H). Moreover, the Kaplan-Meier (KM) curve plot depicted a tendency, with p value = 0.094, that higher levels of circCSPP1 were associated with the faster biochemical recurrence in patients with prostate cancer (Figure S1I).

circCSPP1 promotes prostate cancer *in vitro* and *in vivo*

Two small interference RNAs, si-circCSPP1-1 and si-circCSPP1-2, designed to target the back-splicing junction of circCSPP1, both significantly reduced the expression of circCSPP1 but not the linear form of *CSPP1* (linear-*CSPP1*) in DU145 and PC3 (Figure S2A). We then constructed stable cell lines, including DU145 and PC3, with transfection of lentivirus-circCSPP1 or circCSPP1-sh1/2. qRT-PCR results showed that these lentivirus vectors can only increase/decrease the expression of circCSPP1 rather than the mRNA or protein expression of *CSPP1* in DU145 and PC3 cells (Figures 3A and S2B–S2D). The wound-healing and Transwell assays indicated that overexpression of circCSPP1 promoted migration and invasion of DU145 and PC3 compared with the controls, and vice versa (Figures 3B and 3C). The Cell Counting Kit-8 (CCK-8) and plate colony-formation assays revealed that circCSPP1 overexpression accelerated the proliferation of DU145 and PC3, and vice versa (Figures 3D and 3E). We then injected DU145 cells with lentivirus-circCSPP1 or control subcutaneously on both sides of 6 nude mice, and the result of *in vivo* experiments showed that tumors derived from circCSPP1-overexpression cells grew faster and bigger than the ones in the control group (Figure 3F). Meanwhile, we constructed a xenograft model subcutaneously injected with PC3 cells, including circCSPP1-sh1 cells or negative control (NC) cells, and found that the subcutaneous tumors transfected with circCSPP1-sh1 grew significantly slower than the control group (Figure S2E).

circCSPP1 promotes prostate cancer via augmenting autophagy *in vitro*

To find out in what mechanism cricCSPP1 potentially regulated the progression of prostate cancer cell lines, we carried out the next-generation sequencing of DU145 with the overexpression of circCSPP1 (Figure S3A). The KEGG pathway analysis with differentially expressed genes ($p < 0.01$, log2FC [fold change] $\geq$ 1.5 or log2FC $\leq$ -1.5) showed that circCSPP1 might facilitate prostate cancer progression via regulating autophagy (Figure S3B). Thus, to determine what kind of role circCSPP1 plays in the autophagy process, DU145 and PC3 were both induced into starvation condition by treating with Earle's balanced salt solution (EBSS) at a time gradient of 0 h, 2 h, 4 h, and 6 h, and the results from the qRT-PCR experiments showed that the expression level of circCSPP1 increased over the time gradient (Figure S3C). Then western blotting was conducted, and the results showed that while knocking down circCSPP1 by small interfering RNA (siRNA), the conversion levels of two autophagy-related markers, LC3-II and P62, did not change in DU145 and PC3 if these cells were maintained in complete medium for 8 h with or without bafilomycin A1 (BAF) treatment (Figure S3D). When cultured in EBSS, however, the conversion of LC3-II and P62 in these prostate cancer cell lines treated with BAF increased significantly, compared with the cell lines in dimethyl sulfoxide (DMSO) condition (Figure S3D). In addition, to examine whether blocking autophagy could influence circCSPP1 expression, DU145 and PC3 were treated with BAF at a concentration gradient of 0 μM, 5 μM, and 10 μM, respectively. The qRT-PCR assays showed

circCSPP1 via qRT-PCR in prostate cancer cell lines. (F) FISH of circCSPP1 (red) combined with nuclear DAPI staining (blue) in prostate cancer cell lines. (G) Subcellular distribution of circCSPP1 was detected by nuclear and cytoplasmic separation assay in prostate cancer cell lines. (H) FISH of circCSPP1 (red) combined with nuclear DAPI staining (blue) in DU145 cells with (+) or without (−) RNase R treatment.

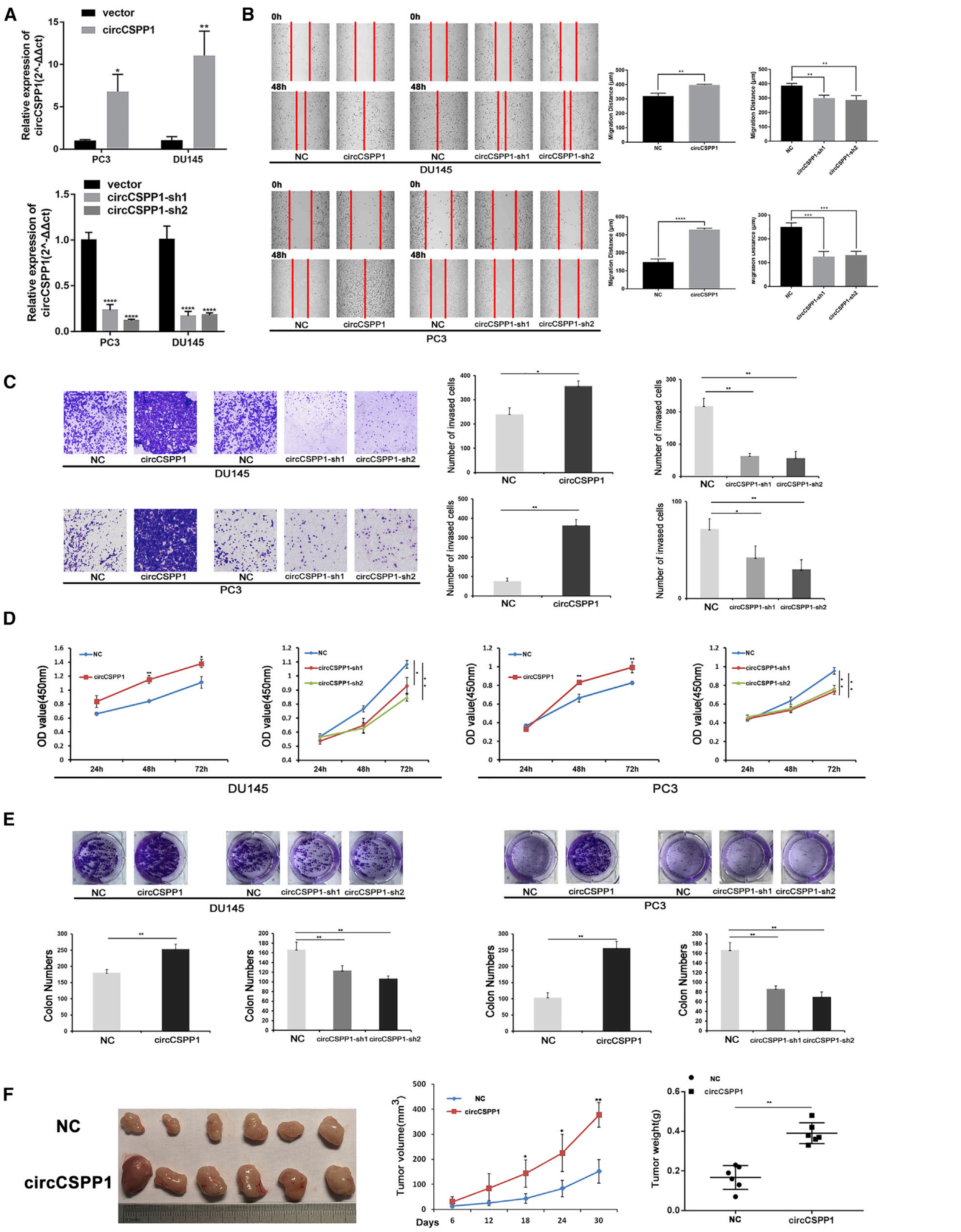

(legend on next page)

that blocking autophagy exerted no effect on circCSPP1 expression (Figure S3E), indicating that circCSPP1 may act as an upstream regulator in autophagy modulation. Then we also confirmed this hypothesis in stable cell lines. Western blotting showed that circCSPP1-sh1/2 also displayed an autophagic inhibition effect, whereas circCSPP1 overexpression remarkably increased the conversion of LC3-II and P62 in DU145 and PC3 cells treated in EBSS (Figures 4A and 4B). Meanwhile, the confocal fluorescence microscopy showed that overexpression/knockdown of circCSPP1 obviously increased/reduced the accumulation of autophagolysosomes in DU145 and PC3 cells (Figure 4C). These results were consistent with the variation of the numbers of autophagosomes exhibited in DU145 and PC3 cells with the overexpression or knockdown of circCSPP1 through TEM (Figure 4D). Subsequently, to exclude the influence of linear CSPP1 on autophagy, we designed 3 siRNAs targeting different sites of *CSPP1* to significantly knock down the expression of CSPP1 at the transcriptional and translational level (Figure S3F), without affecting circCSPP1. Notably, knockdown of CSPP1 had no effect on autophagy in DU145 and PC3 (Figure S3G). Furthermore, to verify the vital role of autophagy induced by circCSPP1 in the promotion of progression in prostate cancer, we carried out a set of cell function assays including CCK-8, plate colony-formation, wound-healing, and Transwell assays using autophagy inhibitors (chloroquine [CQ]). The results revealed that CQ could significantly rescue the proliferation, migration, and invasion that were initially induced by circCSPP1 in DU145 and PC3 cells (Figures S3H–S3K).

Interaction between circCSPP1 and miR-520h

Since circCSPP1 was primarily situated in the cytoplasm, we assumed that it is likely involved in the competing endogenous RNA (ceRNA) network to regulate miRNAs that may be critical to progression of prostate cancer cells. Bioinformatics analyses, including the prediction of target miRNAs for circCSPP1 (circular RNA Interactome) and the detection of autophagy-associated miRNAs (GeneCards and microRNA.org), identified 5 common miRNAs: miR-197-3p, miR-324-5p, miR-375-3p, miR-431-5p, and miR-520h (Figure 5A). The analysis of TCGA and GEO datasets (TCGA_PARD, GSE8126, GSE21036) showed that miR-197-3p, miR-324-5p, and miR-375-3p were upregulated in prostate cancer tissues compared to tumor-adjacent pathologically normal tissues (Figure S4A). A qRT-PCR analysis was performed to further quantify the expression of miR-431-5p and miR-520h in prostate cancer cells, which showed that they were both downregulated (Figure 5B). Nevertheless, the *in vitro* analyses of overexpression/knockdown of circCSPP1 showed that only miR-520h was reduced/elevated accordingly (Figures 5C and 5D). In addition, the RIP experiment with AGO2 antibody, followed by the qRT-PCR assays and agarose gel electrophoresis, confirmed that circCSPP1 was enriched in AGO2-IP analysis, suggesting an AGO2-involved complementary binding between circCSPP1 and target miRNAs (Figure 5E). We then designed the specific biotinylated probe targeting circCSPP1 and found that miR-520h was enriched by circCSPP1 probe by conducting RNA pulldown assay (Figure 5F). Likewise, miRNA pulldown assay showed that biotinylated miR-520h also enriched circCSPP1 (Figure 5G). Subsequently, FISH analysis was employed to study circCSPP1 and miR-520h, suggesting that they were co-localized in the cytoplasm (Figure 5H). Based on the predicted binding sites of miR-520h in the circCSPP1 sequence, dual-luciferase reporter vectors of wild-type (WT) sequences and the mutant (MUT) sequence of circCSPP1 are constructed. The results showed that the luciferase activity of WT reporters was significantly reduced by miR-520h mimics compared with controls (Figure 5I).

circCSPP1 sponges off MiR-520h to promote tumor migration, invasion, proliferation, and autophagy *in vitro* and *in vivo*

To further understand the role of miR-520h and circCSPP1 in promoting prostate cancer progression, we performed rescue experiments, including wound-healing, Transwell, CCK-8, and plate colony-formation assays to investigate the effects of the circCSPP1/miR-520h axis in DU145 and PC3 cells. Wound-healing and Transwell assays showed that circCSPP1 promoted DU145 and PC3 cell migration and invasion, while the miR-520h mimics attenuated this promotion (Figures 6A, 6B, S4B, and S4C). The colony-formation and CCK-8 assays demonstrated that circCSPP1 increased the proliferation ability of DU145 and PC3 cells, while the miR-520h mimics weakened such an association (Figures 6C, 6D, S4D, and S4E). Accordingly, *in vivo* experiments showed that the subcutaneous tumors transfected with circCSPP1 grew significantly faster than control, while the subcutaneous tumors treated with miR-520h grew much slower than the control (Figures 6E–6G). We also conducted a series of rescue experiments to confirm miR-520h's influence on the autophagy activity of prostate cancer cells. The western blotting indicated that both the conversion of LC3-II and the degradation of P62 protein induced by circCSPP1 were abrogated by the miR-520h mimics (Figure 6H). Furthermore, autophagy flux monitoring with sensGFP-stubRFP-LC3 and autophagosomes observation by TEM showed that the number of LC3-II puncta (yellow) and autophagosomes elevated by circCSPP1 were significantly suppressed by the miR-520h mimics (Figures 6I and 6J).

EGR1 is regulated by the circCSPP1/miR-520h axis

We explored whether the circCSPP1/miR-520h axis regulates the expression of autophagy-related genes by targeting their 3′ UTRs. We performed an RNA sequencing (RNA-seq) analysis on 2 groups of DU145 cells transfected with lentivirus-NC and lentivirus-circCSPP1,

Figure 3. circCSPP1 promotes prostate cancer migration invasion and proliferation *in vitro* and *in vivo*

(A) qRT-PCR for circCSPP1 expression with or without lentivirus overexpression and knockdown prostate cancer cell lines. (B and C) The migration and invasion capabilities of DU145 and PC3 cells with circCSPP1 overexpression or knockdown was determined through wound-healing and Transwell assays. (D and E) The proliferative ability of DU145 and PC3 cells with circCSPP1 overexpression or knockdown was determined through the colony-formation and CCK-8 assays. (F) Image of subcutaneous tumors derived from DU145 cells transfected with vector or circCSPP1 in the xenograft model. Tumor volumes were measured every 6 days up to 30 days, and the final tumor weight was calculated. Data are shown as means ± SD. Student's t test, ****$p < 0.0001$, ***$p < 0.001$, **$p < 0.01$, *$p < 0.05$.

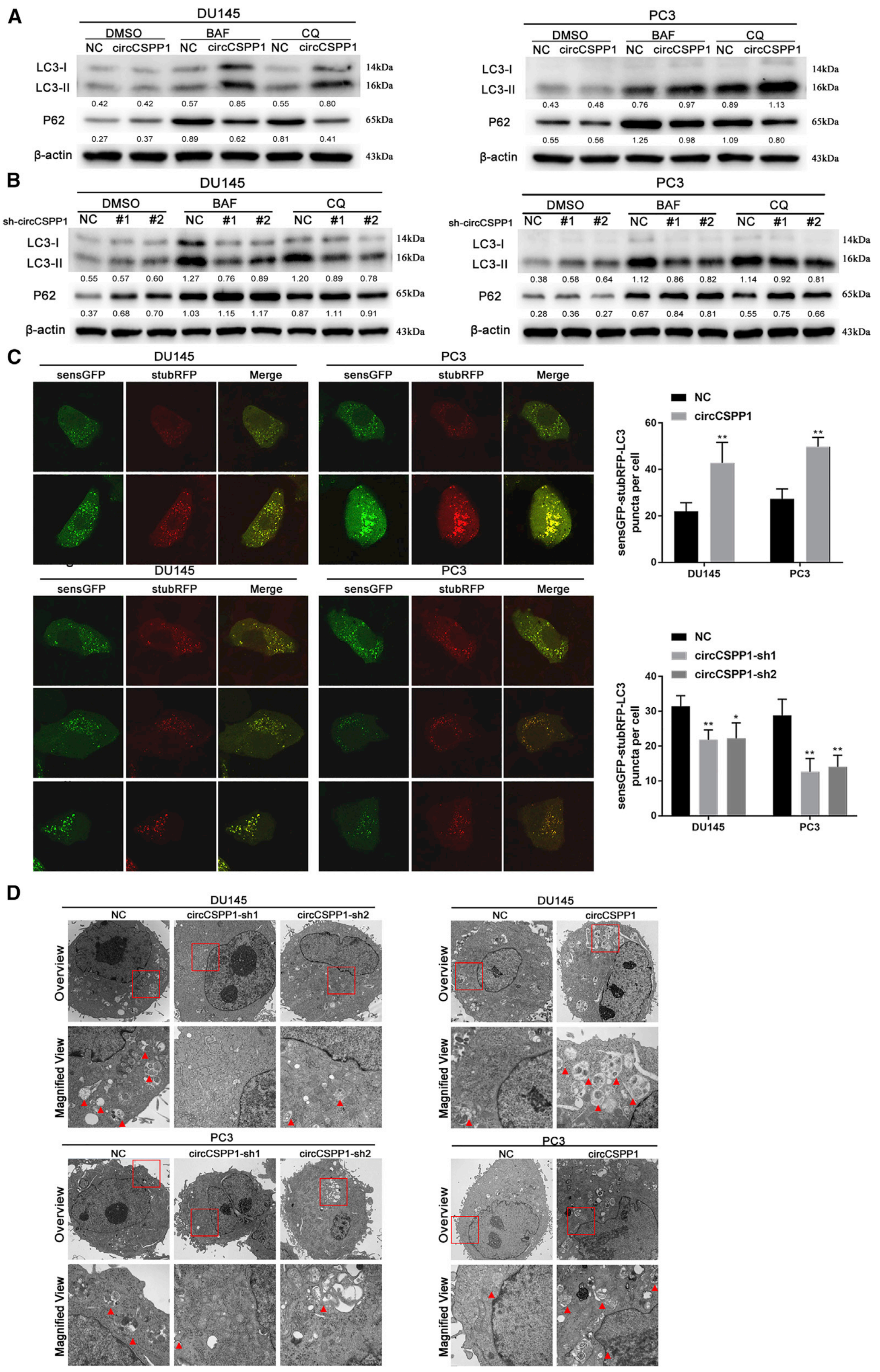

(legend on next page)

each of which contains three replicate samples. The differentially expressed genes are shown in Figure S3A. The bioinformatics algorithms and publicly available datasets, including StarBase, miRWalks, and GeneCards, were used to identify the miR-520h target genes from the differentially expressed genes detected in our RNA-seq data, eventually yielding *EGR1*—an important oncogene for many cancers (Figure 7A). Then we examined the expression of *EGR1* in prostate cancer cell lines with the transfection of miR-520h mimics. As shown in Figures 7B and 7C, both the mRNA and protein expression level of *EGR1* were suppressed by miR-520h mimics or activated by inhibitors compared to the controls. The FISH experiment illustrated that *EGR1* and miR-520h were co-localized in the cytoplasm in PC3 cells (Figure 7D). The *EGR1* luciferase activity of WT reporters was significantly reduced by miR-520h mimics compared to the controls and the mutation group (Figure 7E). We also implemented qRT-PCR to verify the expression of circCSPP1, miR-520h, and *EGR1* both in prostate cancer tissues and benign tissues. The results showed that the expression levels of both circCSPP1 and *EGR1* were higher in the prostate cancer tissues than in the benign tissues, whereas the expression of miR-520h was lower in prostate cancer tissues than that in benign tissues (Figure 7F). The expression level of circCSPP1 was positively correlated with that of *EGR1*, whereas miR-520h exhibited a negative correlation with circCSPP1 or *EGR1* mRNA. We observed the similar correlation patterns in xenograft tumor derived from DU145 cells with circCSPP1 overexpression (Figure 7G).

circCSPP1 promotes progression and autophagy *in vitro* via the miR520h/*EGR1* axis

Cell migration, invasion, proliferation abilities, and autophagy activities were all gauged in prostate cancer cells to investigate whether *EGR1* was targeted and regulated by miR-520h. The results revealed that overexpression of *EGR1* significantly promoted the proliferation, migration, and invasion in prostate cancer cells. However, it was observed respectively in CCK-8, colony-formation, wound-healing, and Transwell assays that introduction of miR-520h mimics rescued these EGR1-related phenotypes. (Figures 8A–8D). Additionally, the western blot showed that overexpression of *EGR1* significantly increased the conversion of LC3-II and P62, and these effects of *EGR1* were also rescued after introducing miR-520h mimics (Figure 8E). Moreover, the results of confocal fluorescent microscopy and TEM showed that miR-520h mimics clearly attenuated EGR1-induced acceleration of autophagy flux and accumulation of autophagosomes (Figures 8F and 8G). Subsequently, to test whether *EGR1* can rescue circCSPP1-induced progression and autophagy in prostate cancer cells, we first designed three siRNAs targeting EGR1, including si-365, si-749, and si-1878. Western blot results showed that si-365 could significantly knock down the expression of *EGR1* in DU145 and PC3 cells (Figure S5A). Then we carried out the rescue experiments and found that knockdown of EGR1 could notably rescue the migration, invasion, and proliferation, which were initially induced by circCSPP1 (Figures S5B–S5E). Additionally, western blot assays showed that the increased conversion of LC3-II and P62 by circCSPP1 can be rescued by knockdown of *EGR1* (Figure S5F). Accordingly, confocal fluorescent and TEM assays showed that downregulating *EGR1* attenuated the activation of autophagy flux and assemble of autophagosomes which were initially induced by circCSPP1 (Figures S5G and S5H).

circCSPP1 is regulated by HnRNP-L

Enlightened by a recent study where HnRNP-L was reported to be responsible for a series of circular RNA biogenesis by alternative splicing in LNCaP, we hypothesized circCSPP1, regulated by HnRNP-L, plays a critical role in promoting aggressive phenotypes in prostate cancer. The differential expression analysis of the dataset GSE72844 showed that circCSPP1 was significantly downregulated when HnRNP-L was knocked down (Figure 9A). Then we constructed prostate cancer cell lines with HnRNP-L overexpression or knockdown through stable or transient transfection. By employing qRT-PCR and western blot, HnRNP-L was significantly downregulated by si-HnRNP-L (Figures S6A and S6B) or overexpressed by lentivirus stably encoding HnRNP-L (Figures S6C and S6D). Further qRT-PCR analysis consequently showed that knockdown or overexpression of HnRNP-L reduced circCSPP1 expression by 60%–70% or upregulated circCSPP1 expression 3–7 times compared to the NC group, respectively. Moreover, the abundance of linear-CSPP1 mRNA did not change while either knocking down or overexpressing HnRNP-L (Figures 9B and 9C). Subsequently, we conducted a RIP assay followed by agarose gel electrophoresis, which unveiled that circCSPP1 bound to HnRNP-L (Figure 9D). Nevertheless, the molecular mechanism of how HnRNP-L regulates the biogenesis of circCSPP1 needs to be further elucidated. Therefore, we rearranged the FASTQ-type data of the RNAs captured by HnRNP-L or nonspecific IgG in the RIP assays, provided in GSE72841.[17] With an advanced analysis of RIP-sequencing data and the transcripts of the corresponding parent gene in the integrative genomic viewer (IGV), we found 5 potential CA-rich motifs of CSPP1 pre-mRNA to be the potential binding sites for HnRNP-L (Figure 9E). These specific motifs contain CA repeats with a variety of lengths. To test whether circCSPP1 is regulated by HnRNP-L through these motifs, we designed 5 specific primers targeting these 5 motifs. The RIP using HnRNP-L antibody and qRT-PCR analysis showed that the motif sequences represented by primers 3 and 4 were successfully amplified (Figures 9F and 9G). To further verify whether these two motifs are the potential sites for both HnRNP-L binding and alternative splicing, we constructed a mini-gene vector by interposing CA-repeat motifs to the flanking introns, on one side or

Figure 4. circCSPP1 promotes prostate cancer autophagy *in vitro*

(A) The overexpression of circCSPP1 promotes autophagy in prostate cancer cell lines, and the conversions of P62, LC3-II, and β-actin under BAF or CQ treatment was detected by western blotting. (B) The knockdown of circCSPP1 attenuates autophagy in prostate cancer cell lines. (C) The overexpression or knockdown of circCSPP1 increases or attenuates the accumulation of LC3-II puncta (green and red overlap). $^{**}p < 0.01$, $^{*}p < 0.05$. (D) Autophagosomes (arrow) observed by transmission electron microscopy (TEM) in circCSPP1 overexpression and knockdown prostate cancer cell lines.

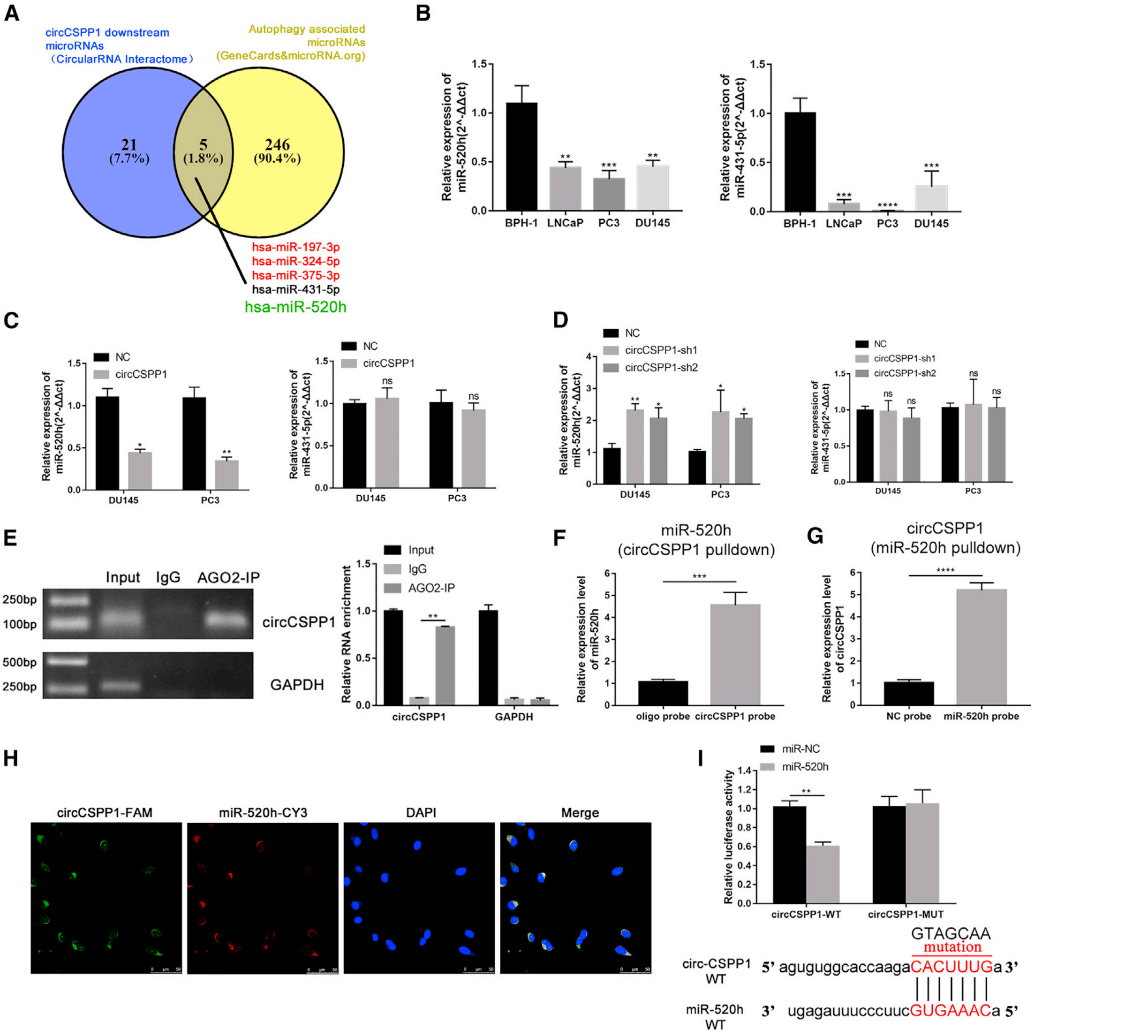

Figure 5. miR-520h is direct target of circCSPP1 in prostate cancer cells

(A) Venn diagram shows the putative miRNAs associated with circCSPP1. (B) Native expression of miR-520h and miR-431-5p were detected via qRT-PCR in prostatic cell lines. (C and D) circCSPP1 is negatively correlated with miR-520h in prostatic cell lines except for miR-431-5p. (E) AGO2 RNA immunoprecipitation (IP) assay shows that circCSPP1 could be captured by AGO2 and was determined by agarose gel electrophoresis (AGE). (F) qRT-PCR analysis of miR-520h in RNA sample by circCSPP1 pull-down. (G) qRT-PCR analysis of circCSPP1 in RNA sample by miR-52h miRNA pull-down. (H) RNA *in situ* hybridization (FISH) detected the co-localization between circCSPP1 (green) and miR-520h (red) in PC3. (I) The interaction of circCSPP1 with miR-520h sequence was predicted by bioinformatics, and the direct target site was confirmed by luciferase reporter assay. Data are shown as means ± SD. Student's t test, ****$p < 0.0001$, ***$p < 0.001$, **$p < 0.01$, *$p < 0.05$.

on each side of GAPDH (Figure 9H). To evaluate the efficiency in forming GAPDH circRNA catalyzed by HnRNP-L, the mini-gene constructs were transfected into HEK293T cells followed by qRT-PCR assays. The results showed that these CA-repeat motifs inserted to the flanking introns remarkably enhanced GAPDH circRNA formation, especially when the insertion occurred on both sides (Figure 9I). Meanwhile, knocking down HnRNP-L significantly decreased the GAPDH circRNA abundance in HEK293T cells (Figure 8J). In conclusion, these data demonstrated that the high level of circCSPP1 could be an outcome of upregulation by HnRNP-L-involved alternative splicing.

HnRNP-L positively regulated autophagy in prostate cancer cells

Our previous studies have proved that HnRNP-L promotes the malignant progression in prostate cancer and is correlated positively with the pathological stages,[18] consistent with the current findings that HnRNP-L facilitated proliferation and metastasis in prostate cancer

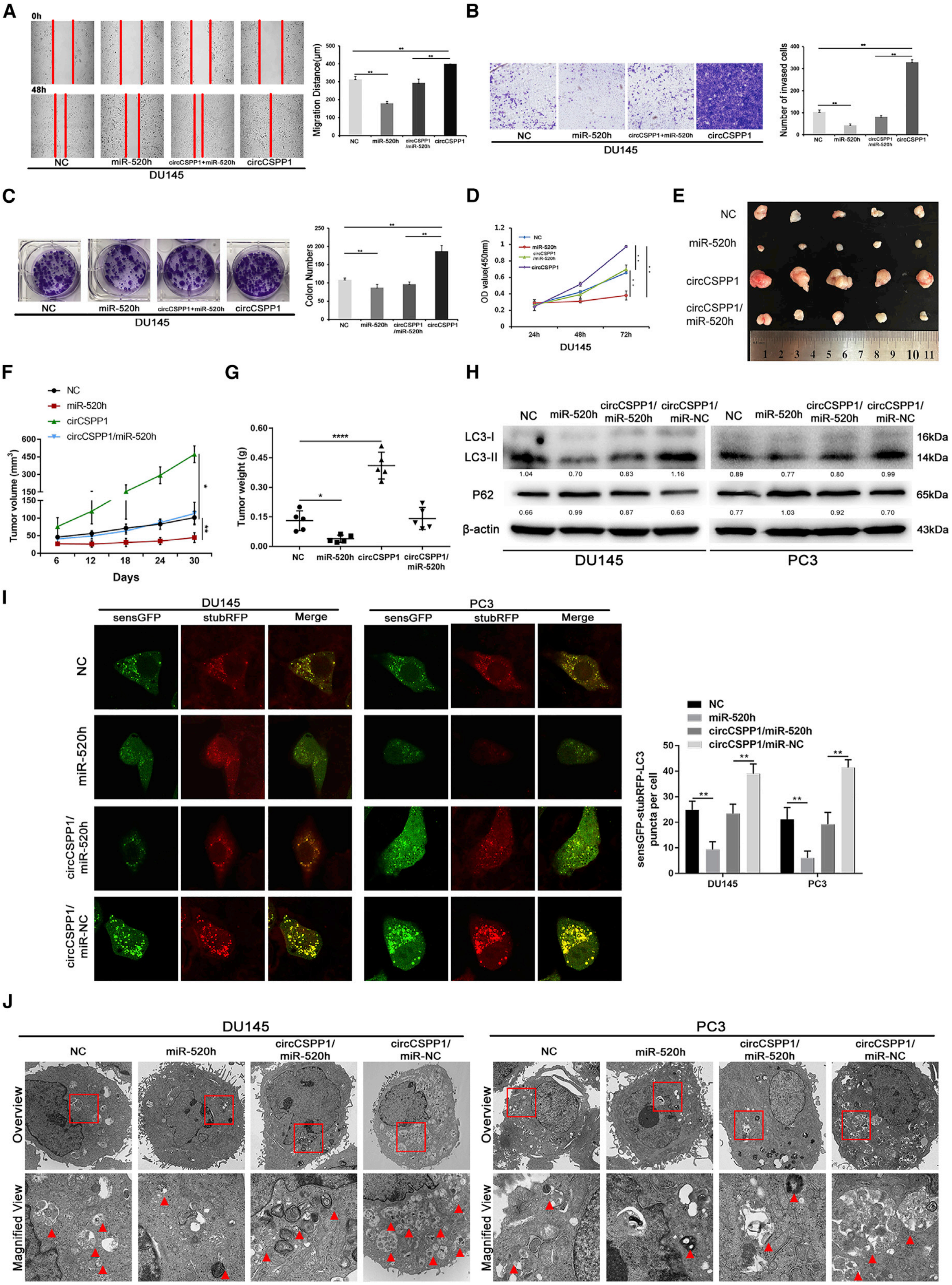

(legend on next page)

cell lines by regulating circCSPP1. Furthermore, we also confirmed that HnRNP-L positively regulated autophagy in prostate cancer. As shown in Figures S7A and S7B, overexpression or silence of HnRNP-L could significantly increase or decrease LC3-I/LC3-II conversion and attenuated or accelerated P62 degradation. Consistently, when upregulating or knocking down HnRNP-L, the autophagy flux was enhanced or suppressed (Figures S7C and S7D), and the autophagosomes were accumulated or reduced (Figure S7E).

DISCUSSION

circRNAs have drawn increasing attention from the research field of non-coding RNAs. Dysregulations in circRNAs have been reported in a variety of cancers, including melanoma,[22] bladder cancer,[23] hepatocellular carcinoma,[24] and glioma.[25] Although circCSPP1 was recently reported to be associated with tumor proliferation, invasion, and migration in ovarian cancer[26] and colorectal carcinoma,[27] its circular structure has not been confirmed in these studies. In this study, we used a combination of RNase R test, divergent primers, convergent primers, and Sanger sequencing to definitively demonstrate the circularization of exon #8 with exon #11 in the parent gene *CSPP1*. Survival analysis revealed that circCSPP1 might correlate with shorter BCR in patients with prostate cancer, even though the p value = 0.094. We surmised that insufficient sample size might be a factor. Thus, more sequencing datasets of circular RNA in prostate cancer either in public databases or in our own patient cohort were needed to reevaluate the association between circCSPP1 and BCR in prostate cancer. It was previously reported that *CSPP1* has two primary isoforms: CSPP and CSPPL.[28] Despite previous studies indicating that the inhibition of CSPP/CSPPL could induce G1 phase arrest in the cell cycle and the destabilization of desmosomes,[29,30] how *CSPP1* functions in prostate cancer remains unclear. The expression of linear CSPP1 did not change significantly in the circCSPP1 overexpressed/knockdown cell lines, indicating that we can focus on the function investigation on circCSPP1 without considering the effects of linear CSPP1. The study showed that the overexpression of circCSPP1 strengthened autophagy, which induced tumor progression, and this phenomenon became more apparent after EBSS starvation, and vice versa. Intriguingly, it was overexpression of circCSPP1 rather than linear CSPP1 that promoted autophagy.

Mounting evidence has shown that non-coding RNAs, including lncRNAs and miRNAs, were involved in autophagy-related tumor progression;[31,32] however, circRNAs have been rarely reported to relate to either autophagy or cancer progression, especially for prostate cancer. Previous research indicated that circRNAs play a critical role in various cellular functions, including competing endogenous RNA (ceRNA), translating peptides, regulating transcription, acting as a scaffold for protein complexes, and so on.[10] This study focused on deciphering the mechanistic relationship between circCSPP1, autophagy, and tumor progression in prostate cancer. According to our data, circCSPP1 was mainly localized in the cytoplasm, and AGO2 RIP showed that circCSPP1 was specifically bound to AGO2, suggesting circCSPP1 acted as a miRNA sponge. Bioinformatics analysis was used to search candidate miRNAs that bind to circCSPP1; as a result, miR-520h was identified as the downstream target of circCSPP1.

The analysis based on publicly available datasets as well as our own patient samples indicated that miR-520h inhibited not only autophagy but also tumor proliferation, migration, and invasion in prostate cancer. The FISH and dual-luciferase reporter assays showed that miR-520h is a direct target of circCSPP1. The bioinformatics analysis of multiple relevant datasets, shown in the Venn diagram in Figure 7A, indicated that *EGR1* is the only overlapping gene (or mRNA). We further confirmed that *EGR1* was a direct target of miR-520h by the qPCR, FISH, and dual-luciferase reporter assays. The rescue experiment using miR-520h mimics indicated that *EGR1* can promote cell vitality, migration, and invasion by inducing autophagy, and such an effect may be partially countered by miR-520h. Finally, we verified the association among the circCSPP1/miR-520h/EGR1 axis in both our prostate cancer samples and cancerous xenografts. It has been reported that *EGR1* regulated autophagy by transcriptionally affecting autophagy-associated gene expression, including LC3B.[19] Further investigation is warranted to identify other important autophagy-associated genes regulated by *EGR1* in prostate cancer.

Back-splicing, a brand-new branch of alternative splicing, gets involved in the formation of circRNAs when occurring in pre-mRNAs;[10] however, the complete process of the biogenesis of circRNAs is not clear yet. Interestingly, circCSPP1 was remarkably downregulated by si-HnRNP-L (GSE72844),[17] suggesting that HnRNP-L is a critical factor in this alternative splicing and regulates circRNA formation. In the study, the data of the RIP with HnRNP-L antibody depicted that the pre-RNA of circCSPP1 contains five CA-rich regions, two of which are highly likely to be the binding sites of HnRNP-L. We further verified these two CA-rich binding sites in the flanking introns upregulating circCSPP1 via the mini-gene system. Although these experiments

Figure 6. circCSPP1 sponges miR-520h to promote prostate cancer migration, invasion, proliferation, and autophagy *in vitro* and *in vivo*

(A and B) The migration and invasion capabilities of DU145 transfected with lentivirus-circCSPP1 and/or miR-520h mimics were determined with the wound-healing and Transwell assays. (C and D) The proliferative ability of DU145 transfected with lentivirus-circCSPP1 and/or miR-520h mimics was determined through the colony-formation and CCK-8 assays. (E–G) Image of subcutaneous tumors derived from DU145 cells transfected with lentivirus-circCSPP1 and/or miR-520h mimics in the xenograft model. Tumor volumes were measured every 6 days up to 30 days, and the final tumor weight was calculated. (H) The conversion of P62, LC3-II normalized to β-actin in DU145, and PC3 transfected with lentivirus-circCSPP1 and/or miR-520h mimics under BAF treatment were detected by western blotting. (I) The accumulation of LC3-II puncta (yellow, green, and red overlap) was detected in DU145 and PC3 transfected with circCSPP1-overexpressing lentivirus or miR-520h mimics or co-transfected with both circCSPP1-overexpressing lentivirus and miR-520h mimics. (J) Autophagosomes (arrow) were observed by TEM in DU145 and PC3 transfected with lentivirus-circCSPP1 and/or miR-520h mimics. Data are shown as means ± SD. Student's t test, ****$p < 0.0001$, ***$p < 0.001$, **$p < 0.01$, *$p < 0.05$.

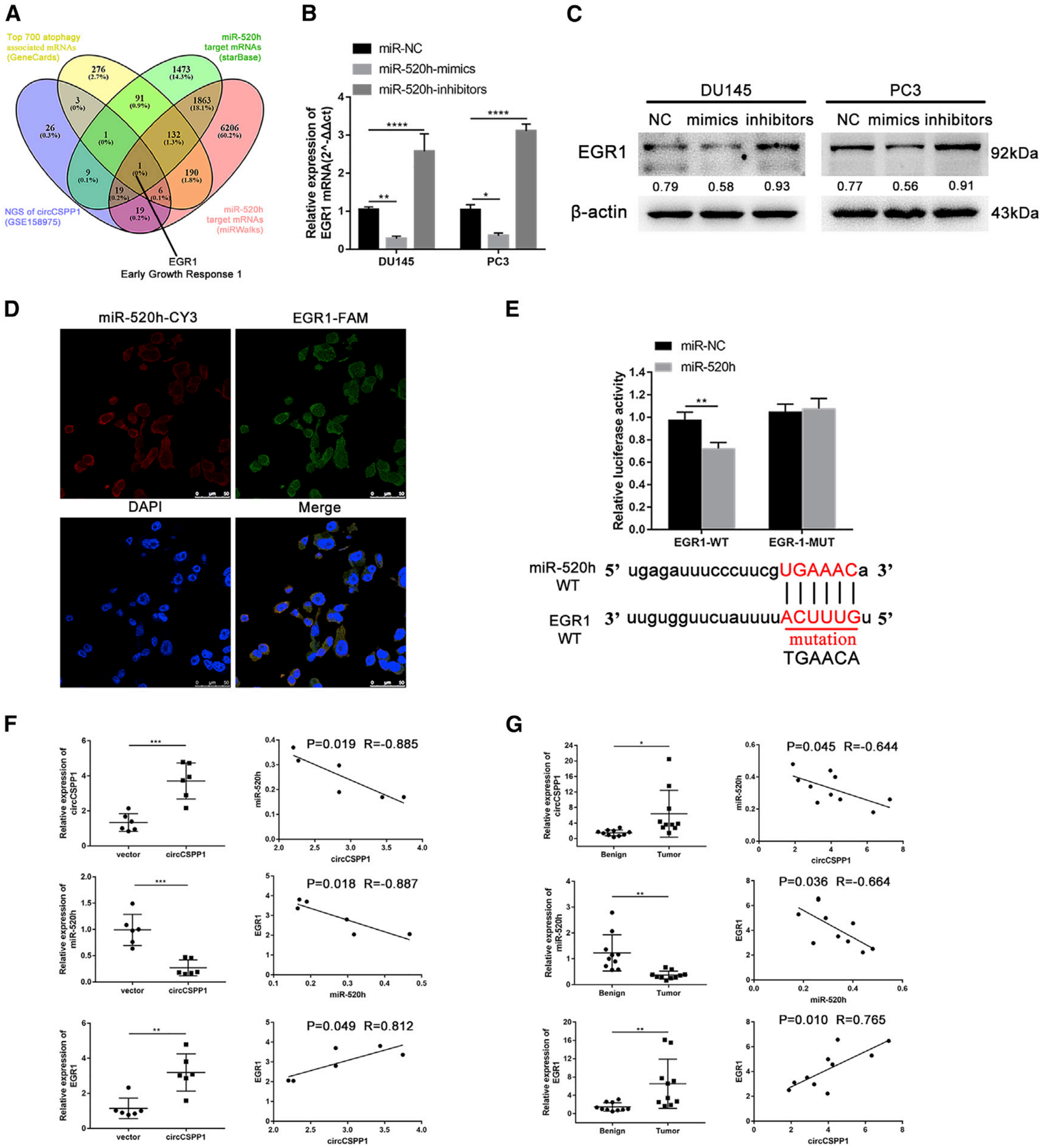

Figure 7. *EGR1* is direct target of miR-520h in prostate cancer cells

(A) Venn diagram shows *EGR1* is the only gene in the overlap. (B) The mRNA expression level of *EGR1* was downregulated or upregulated in DU145 and PC3 transfected with miR-520h mimics or inhibitors. (C) The protein expression level of *EGR1* was downregulated or upregulated in DU145 and PC3 transfected with miR-520h mimics or inhibitors. (D) RNA *in situ* hybridization detected the co-localization between *EGR1* (green) and miR-520h (red) in PC3 cells. (E) The interaction of miR-520 with *EGR1* gene sequences was predicted by bioinformatics, and dual-luciferase reporter assays were performed to confirm their direct target sites. $^{**}p < 0.01$. (F and G) Pattern of the expression of circCSPP1/miR-520h/*EGR1* normalized to that of β-actin and the Pearson correlation analysis between any two of three above in 10 pairs of human prostate cancer and benign tissues or 6 pairs of xenograft tumors with vector or circCSPP1 overexpression. Data are shown as means ± SD. Student's t test, $^{***}p < 0.001$, $^{**}p < 0.01$, $^{*}p < 0.05$.

were performed *in vitro*, other studies, such as the CRISPR-Cas9 mouse experiments or fluorescence resonance energy transfer (FRET) assay, are still needed for further validation.

In summary, our study illustrated the association between HnRNP-L, circCSPP1, miR-520h, and *EGR1* in prostate cancer and the potential underlying mechanism to promote malignancy. Overexpression of HnRNP-L upregulates the circCSPP1/miR-520h/*EGR1* axis, then inducing autophagy and eventually accelerating prostate carcinoma progression. Above all, our study provides new insights to the role of circRNAs in regulating autophagy in prostate cancer.

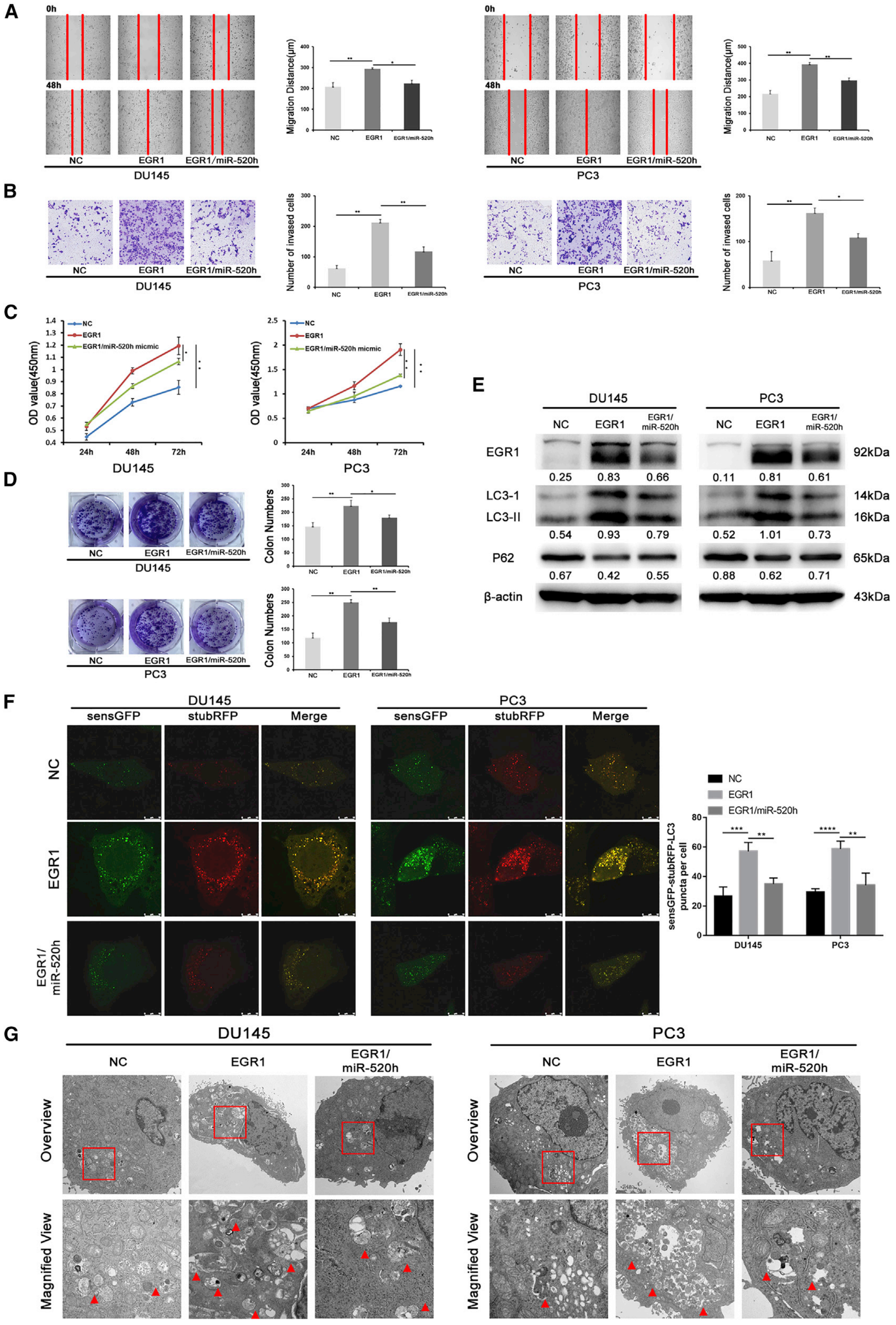

(legend on next page)

MATERIALS AND METHODS

Ethics statement

This study was approved by Ethics Committee of Zhujiang Hospital, Southern Medical University. The informed consents were signed by all the patients. According to the ethical and legal standards, every specimen was made and handled anonymously. All animal experiments in this study were carried out following the guidelines of the Institute for Laboratory Animal Research at Southern Medical University, Guangzhou, P.R. China.

Patient samples

Prostate cancer tissues and benign prostate hyperplasia tissues were respectively collected from 10 patients of the Cancer Center of Guangzhou Medical University (Guangzhou, China) between 2018 and 2020. All the clinical and pathological information are summarized in Table S2 and S3. Fresh tissues were viewed and approved by two pathologists, frozen immediately in liquid nitrogen, and stored at -80°C.

Cell culture

All the human prostate cancer cell lines including PC3, DU145, LNCaP, and benign prostate hyperplasia cell line BPH-1, and human embryonic kidney cells (293T) were obtained from the Cell Bank of the Chinese Academy of Sciences (Shanghai, China). According to the instructions, PC3 was cultured in F12K medium (Procell), DU145 was cultivated in DMEM medium (Gibco), and 293T, LNCaP, and BPH-1 were maintained in RPMI-1640 medium (Gibco). All media listed above were supplemented with 10% fetal bovine serum (FBS, Gibco) and 1% penicillin-streptomycin (Gibco). All cell lines were maintained at 37°C with 5% CO_2. For the autophagy induction experiments, all stable or transient transfected cell lines were maintained in EBSS in the presence of 10 μM BAF and/or 50 nM CQ for 8 h. DMSO was used as NC.

Transfection

The hsa_miR-520h mimics/inhibitors, pcDNA3.1-*EGR1*, and siRNAs targeting circCSPP1, *EGR1*, or HnRNP-L were synthesized and transfected into cell lines using siRNA-Mate or GP-transfect-Mate (Genepharm) following the manufacturer's instruction. Lentivirus vectors encoding HnRNP-L, sh-/circCSPP1, sensGFP-stubRFP-LC3, and *AGO2* were constructed and transfected into prostate cancer cell lines with Hitrans-GP (GeneChem). All the lentiviral transfected cells were treated with 1 μg/mL puromycin for 7 days to generate the stable cell lines. siRNAs and miR-520 mimic/inhibitor sequence details are shown in Table S1.

RNA extraction and real-time PCR

Total RNA was isolated from cells by using TRIzol reagent (Takara, Tokyo, Japan) according to the instructions of the manufacturer. Nuclear and cytoplasmic RNA fractions were separated from 10^2–10^7 cell pellets with the PARIS Kit (Ambion, Life Technologies) according to the manufacturer's instructions. RNA sample was treated with RNase R (Geneseed) at 37°C for 30 min to obtain purified circRNA. For circRNA and mRNA, cDNA was reverse-transcribed by using HiScript II Q RT SuperMix for qPCR (R223-01, Vazyme). For miRNA, cDNA was synthesized by using PrimeScript RT Reagent Kit with gDNA Eraser (RR0471, Takara) with Bulge-Loop miRNA RT Primer (R10031.7, RiboBio). qRT-PCR was carried out using the SYBR Green Realtime PCR Master Mix (QPK-201, TOYOBO) with the CFX connect qPCR Detection System (Bio-Rad). β-actin was used as the endogenous control for mRNA and circRNAs, while U6 was used for microRNAs to calculate the relative fold changes for transcript abundance. Every experiment was carried out in three replicates. Primers sequence details are shown in Table S1.

Nucleic acid electrophoresis

The cDNA was augmented by qRT-PCR, and gDNA was amplified using 2× Taq PCR MasterMix by T100 Thermal Cycler (Bio-Rad). Then all types of PCR products were separated by 2% agarose gel electrophoresis with TAE running buffer. The electrophoresis was run at 110 V for 40 min. Finally, the gels were irradiated by ultraviolet rays, and the targeted gene bands were measured by comparing with the DNA marker DL2000 (3427A, Takara). Every experiment was carried out in three replicates.

Western blot analysis

For detecting relative autophagy markers LC3 and P62, cancer cells were treated with EBSS containing 10 μM BAF or 50 nM CQ for 8 h before being lysed by RIPA buffer with PMSF on ice for 15 min. Then the cell lysis was mixed with 5× protein loading buffer and subsequently denaturalized at 100°C for 10 min. Total protein denaturants were separated by SDS-PAGE, transferred onto polyvinylidene fluoride (PVDF) membranes (Millipore), and blocked with 5% skim milk in Tris-buffered saline with 0.1% Tween® 20 detergent for 1 h. The membranes were incubated with primary antibodies against β-actin (BA2305, Boster), LC3A (NB100-2331, Novus), SQSTM1/P62 (sc-28359, Santa Cruz), HnRNP-L (ab6106, Abcam), and *EGR1* (#4154 CST) at 4°C overnight. Then, all the membranes were immersed in horseradish peroxidase-linked secondary antibodies against rabbit or mouse IgG. Every experiment was carried out in three replicates. The bands were visualized using

Figure 8. miR-520h silences *EGR1* to inhibit prostate cancer migration, invasion, proliferation, and autophagy *in vitro*

(A and B) The migration and invasion capabilities of DU145 and PC3 transfected with pcDNA3.1-*EGR1* and/or miR-520h mimics were determined with the wound-healing and Transwell assays. (C and D) The proliferative ability of DU145 and PC3 transfected with pcDNA3.1-*EGR1* and/or miR-520h mimics was determined through the colony-formation and CCK-8 assays. (E) *EGR1* expression and the conversion of P62, LC3-II normalized to β-actin in DU145 and PC3 transfected with pcDNA3.1-*EGR1* and/or miR-520h mimics under BAF treatment were detected by western blotting. (F) The accumulation of LC3-II puncta (yellow, green, and red overlap) was detected in DU145 and PC3 transfected with pcDNA3.1-*EGR1* and/or miR-520h mimics. (G) Autophagosomes (arrow) were observed by TEM in DU145 and PC3 transfected with pcDNA3.1-*EGR1* and/or miR-520h mimics. Data are shown as means ± SD. Student's t test, ****$p < 0.0001$, ***$p < 0.001$, **$p < 0.01$, *$p < 0.05$.

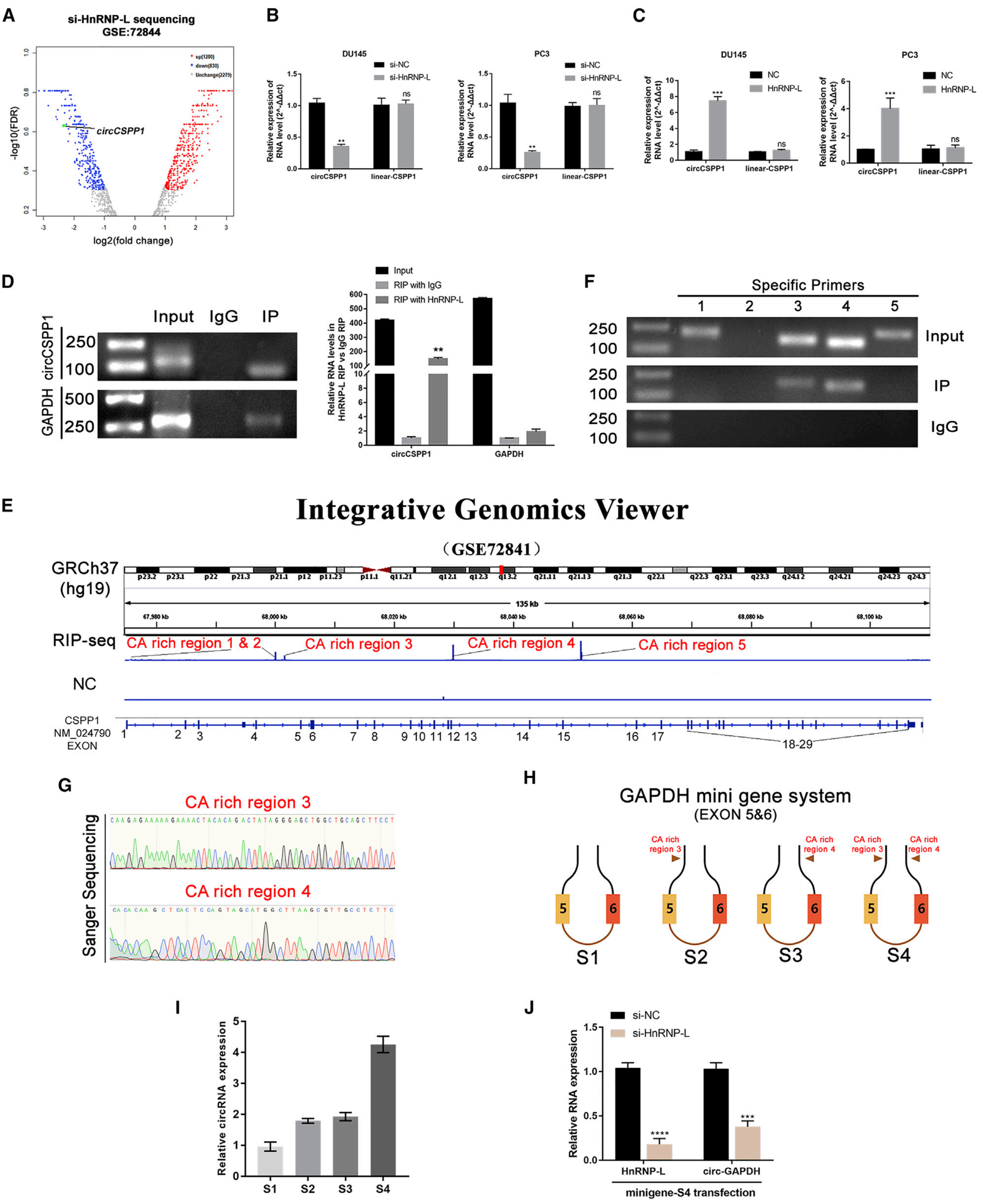

(legend on next page)

chemiluminescence imaging system (CLiNX ChemiScope Touch, Shanghai) and quantified by ImageJ software.

RIP assay

2×10^7 DU145 cells were collected and lysed by ice-cold polysome lysis buffer with protease inhibitor and RNase inhibitor from the RNA Immunoprecipitation Kit (Bes5101, BersinBio). The major part (90%) of the cell lysis was incubated with anti-HnRNP-L or AGO2 (IP group) and non-specific IgG (IgG group), respectively, on vertical mixer at 4°C for 16 h, while the other was kept as an input group. Subsequently, two groups were mixed with protein A/G beads by vortex at 4°C for 1 h, followed by recovery of beads and RNA elution from the mixture. The RNA samples among IP, IgG, and input groups were respectively quantified using NanoDrop™ Spectrophotometers (ND2000USCAN, Thermo Scientific™), followed by qRT-PCR analysis and electrophoresis.

RNA pulldown assay

The biotinylated probes targeting circCSPP1, biotinylated miR-520h mimics, and oligo probes were all synthesized by Genepharm. Briefly, 100 μg total RNA from DU145 cells was collected using the traditional TRIzol method. Then 200 μmol biotinylated probes targeting circCSPP1 or biotinylated miR-520h mimics was mixed well with 500 μg streptavidin magnetic beads, followed by adding to the RNA sample, and the mix was rotated for 30 min at 37°C. Subsequently, elution buffer was added to the mixtures, and the pulldown RNA products were extracted and validated by qRT-PCR for detection of miR-520h or circCSPP1. The probes used in the experiment are shown in Table S1.

FISH

1×10^4 cells were embedded to a cover slide in a 48-well plate and cultured overnight. Prostate cancer cells were fixed by 4% paraformaldehyde for 15 min at room temperature after being washed with 1× phosphate-buffered saline (PBS) for 5 min × 2 and then penetrated with 0.1% Triton X-100. Subsequently, cells were washed with 1× PBS for 5 min × 2 and treated with 2× saline sodium citrate (SSC) for 30 min at 37°C. The probes (Genepharm) targeting circCSPP1/*EGR1*/hsa_miR-520h were pre-mixed with hybridization buffer and denatured at 73°C for 5 min. After hybridization, the slides were washed with 0.1% Tween 20 for 5 min at 42°C and then washed with 2× SSC at 42°C for 5 min × 2. DAPI was re-dyed in the dark at room temperature for 20 min and washed with 1× PBS, 5 min × 2. After treatment with antifade reagent, the cell slides were adhered to the slide (face up) with neutral gum before observation under a fluorescence microscope. The probes used in the experiment are shown in Table S1.

Luciferase reporter assay

Dual-luciferase reporter vector pmirGLO (Promega) was used for the luciferase assays. circCSPP1/EGR-1 WT and circCSPP1/EGR-1 MUT reporter vectors were constructed and inserted into the pmirGLO. 5×10^5 cells were seeded into a 12-well plate and cultured for 24 h at 37°C with 5% CO_2. Subsequently, 1.6 μg reporter plasmids (circCSPP1/*EGR1* WT, circCSPP1/*EGR1* MUT) together with 20 μM hsa_miR-520h mimics or NC were transfected into DU145/PC3 cells. Then the transfected cells were transferred to the incubator and cultured for another 48 h. Finally, the Dual-Luciferase Reporter System Kit (Promega) was used to detect the luciferase activity with Tecan M1000 microplate reader.

Electron microscopy

Adherent cells estimated at 1×10^6 were treated with 0.25% trypsin for only 30 s to keep cell membranes intact. Cell suspension was centrifuged at 800 rpm for 5 min, followed by supernatant removal. Each sample was fixed with 2% glutaraldehyde at 4°C for over 15 min and washed with PBS three times for 10 min each. Samples were post-fixed with 1% OsO_4 followed by an ascending gradient dehydration step of ethanol and infiltration with propylene oxide. After ultrathin sectioning and staining with 3% lead citrate-uranyl acetate, samples were observed under an electron microscope (HT-7800, Hitachi High-tech).

Examination of autophagy flux

PC3 and DU145 cells were cultured and transfected with lentivirus carrying sensGFP-stubRFP-LC3 at 37°C for 48 h. Next, the transfected cells were treated with EBSS containing 50 μM BAF for 8 h. Cells were fixed with 4% paraformaldehyde for 30 min. Finally, the autophagy flux was analyzed using a confocal fluorescence microscopy (Leica, Germany). In merged images, yellow spots represent autophagosomes, while red spots represent autolysosomes.

Cell proliferation and colony-formation assays

Cell proliferation was determined using CCK-8 assays (MA0218-5, Meilunbio). The transfected cells were seeded into 96-well plates at a density of 3,000 cells per well. 100 μL complete medium containing 10 μL CCK-8 reagent was added into each well at 0, 24, 48, 72, and 96 h after seeding. All plates were scanned using a microplate reader (Bio-Rad) in another 2 h incubation. The absorbance at 450 nm was measured and analyzed. Prostate cancer cells were plated at an initial

Figure 9. circCSPP1 is upregulated by HnRNP-L

(A) The volcano plot shows circCSPP1 was downregulated in LNCaP cell with HnRNP-L downregulation. (B and C) circCSPP1 is positively correlated with HnRNP-L in prostate cancer cell lines via qRT-PCR. Student's t test, ***p < 0.001, **p < 0.01; ns, not significant. (D) RIP shows circCSPP1 is binding with HnRNP-L directly; **p < 0.01. (E) RIP-sequencing shows the potential binding sites between circCSPP1 with HnRNP-L. (F) RIP-qPCR with specific primers presents that the 3rd and 4th CA repeat sequences of pre-circCSPP1 are the direct binding sites with HnRNP-L. (G) Sanger sequencing confirmed the binding sites in the pre-mRNA of CSPP1 with HnRNP-L. (H) Schematic description showing 4 designed scenarios of GAPDH minigene construction. (I) Relative circRNA expression standardized by pre-mRNA levels in different GAPDH minigenes was analyzed by qRT-PCR. (J) qRT-PCR was performed to evaluate circular GAPDH formation in S4 minigene-transfected cells with the knockdown of HNRNP-L. Student's t test, ****p < 0.0001, ***p < 0.001.

density of 500 cells per well in a 6-well plate and cultured at 37°C with 5% CO_2 for 14 days. Then, the colonies were fixed for 20 min with 4% paraformaldehyde and stained for 15 min with crystal violet. After discarding the staining solution, the plates were air-dried at room temperature and then observed under the light microscope. Every experiment was carried out in three replicates.

Migration and invasion assay

Wound-healing assays were carried out to evaluate the migration ability of prostate cancer cells. Transfected cells were seeded in 6-well plates at a density of 1×10^6 cells/well and grown to 90%–100% in 10% FBS medium. Then linear wounds were scratched with a sterile 200 μL plastic pipette tip in each well, and PBS was used to remove the detached cells. Cells were cultured in FBS-free medium to inhibit cell proliferation. Images of the scratched area were captured at indicated times (0 and 24 h) using a Leica light microscope. For invasion assay, the Transwell chamber was precoated with Matrigel matrix (BD, 356234), and 6×10^4 cells were seeded to the upper chamber. Subsequently, 500 μL of DMEM medium containing 10% FBS was added to the lower chamber. The cells on the top surface were removed with a cotton ball after incubation for 24 h, and the cells that invaded to the lower membrane surface were fixed with 4% paraformaldehyde and stained with 0.1% crystal violet solution. The invaded cells were then photographed and counted under an inverted microscope. Every experiment was carried out in three replicates.

Animal experiments

The 4-week-old BALB/c nude mice (male) were obtained from the Guangdong Experimental Animal center (Guangzhou, China). The male BALB/c nude mice were subcutaneously injected with 3×10^6 stably transfected DU145 cells (empty vector or circCSPP1-overexpression) or PC3 cells (empty vector or circCSPP1-knockdown) in both back sides. For the rescue experiments, the mice transfected with vector or circCSPP1 stable-expressed DU145 cells were randomly divided into two groups, half of which were injected with miR-NC and the other with miR-520h mimics intratumorally every 3 days for 2 weeks. The growth of implanted prostate cancer tumors was monitored by measuring their volume every 6 days. Finally, the mice were sacrificed, and their xenografts were measured and photographed.

Statistical analysis

All data are shown as mean ± SD processed by GraphPad Prism 7.0 (La Jolla, CA, USA). Student's t test analysis was used to evaluate the normalized data. Pearson correlation assay was used to analyze expression correlation (circCSPP1, miR-520h, and *EGR1*). The Kaplan-Meier method was used to estimate the OS and BCR curves. All statistical tests were two-sided and considered statistically significant when p values were less than 0.05.

Availability of data and materials

The RNA-seq data of stable prostate cancer cell lines (DU145-vector; DU145-circCSPP1) analyzed during this study has been deposited in NCBI's Gene Expression Omnibus (https://www.ncbi.nlm.nih.gov/geo/query/acc.cgi?acc=GSE158975) and is also available in the supplemental information files.

SUPPLEMENTAL INFORMATION

Supplemental information can be found online at https://doi.org/10.1016/j.omtn.2021.10.006.

ACKNOWLEDGMENTS

This research was supported by grants from the National Natural Science Foundation of China (81773277, 82003271, 82072813, and 82173039), the China Postdoctoral Science Foundation (2019M662979, 2018M643126, and 2019M662865), the Guangzhou Science and Technology Project (201803010014, 201803040001), the Guangdong Basic and Applied Basic Research Foundation (2019A1515110033 and 2021A1515010659), and the Guangdong Medical Science Research Project (A2020544).

AUTHOR CONTRIBUTIONS

X.M. and J. Lu conceived of the study, and C.Z. carried out its design. J. Lu, C.Z., and F.S. performed the experiments. D.L., X.T., S.W., K.W., T.Y., and Weibo Zhong collected clinical samples. J. Lu, D.L., Y.L., B.W., and Y.C. analyzed the data. Y.Z. provided administrative or technical support. J. Lu, C.Z., Weide Zhong, and Z.J. wrote the paper. All authors participated in writing the manuscript and approved the final version.

DECLARATION OF INTERESTS

The authors declare no competing interests.

REFERENCES

1. Siegel, R.L., Miller, K.D., and Jemal, A. (2020). Cancer statistics, 2020. CA Cancer J. Clin. *70*, 7–30.
2. Bray, F., Ferlay, J., Soerjomataram, I., Siegel, R.L., Torre, L.A., and Jemal, A. (2018). Global cancer statistics 2018: GLOBOCAN estimates of incidence and mortality worldwide for 36 cancers in 185 countries. CA Cancer J. Clin. *68*, 394–424.
3. Cheng, H.H., Sokolova, A.O., Schaeffer, E.M., Small, E.J., and Higano, C.S. (2019). Germline and Somatic Mutations in Prostate Cancer for the Clinician. J. Natl. Compr. Canc. Netw. *17*, 515–521.
4. Dong, X., and Chen, R. (2020). Understanding aberrant RNA splicing to facilitate cancer diagnosis and therapy. Oncogene *39*, 2231–2242.
5. Santanam, U., Banach-Petrosky, W., Abate-Shen, C., Shen, M.M., White, E., and DiPaola, R.S. (2016). Atg7 cooperates with Pten loss to drive prostate cancer tumor growth. Genes Dev. *30*, 399–407.
6. Howard, N., Clementino, M., Kim, D., Wang, L., Verma, A., Shi, X., Zhang, Z., and DiPaola, R.S. (2019). New developments in mechanisms of prostate cancer progression. Semin. Cancer Biol. *57*, 111–116.
7. Anastasiadou, E., Jacob, L.S., and Slack, F.J. (2018). Non-coding RNA networks in cancer. Nat. Rev. Cancer *18*, 5–18.
8. Hua, J.T., Chen, S., and He, H.H. (2019). Landscape of Noncoding RNA in Prostate Cancer. Trends Genet. *35*, 840–851.
9. Liu, J., Li, D., Luo, H., and Zhu, X. (2019). Circular RNAs: The star molecules in cancer. Mol. Aspects Med. *70*, 141–152.
10. Kristensen, L.S., Andersen, M.S., Stagsted, L.V.W., Ebbesen, K.K., Hansen, T.B., and Kjems, J. (2019). The biogenesis, biology and characterization of circular RNAs. Nat. Rev. Genet. *20*, 675–691.

11. Chen, B., and Huang, S. (2018). Circular RNA: An emerging non-coding RNA as a regulator and biomarker in cancer. Cancer Lett. *418*, 41–50.

12. Sveen, A., Kilpinen, S., Ruusulehto, A., Lothe, R.A., and Skotheim, R.I. (2016). Aberrant RNA splicing in cancer; expression changes and driver mutations of splicing factor genes. Oncogene *35*, 2413–2427.

13. Robinson, T.J., Freedman, J.A., Al Abo, M., Deveaux, A.E., LaCroix, B., Patierno, B.M., George, D.J., and Patierno, S.R. (2019). Alternative RNA Splicing as a Potential Major Source of Untapped Molecular Targets in Precision Oncology and Cancer Disparities. Clin. Cancer Res. *25*, 2963–2968.

14. Cai, L., Tsai, Y.H., Wang, P., Wang, J., Li, D., Fan, H., Zhao, Y., Bareja, R., Lu, R., Wilson, E.M., et al. (2018). ZFX Mediates Non-canonical Oncogenic Functions of the Androgen Receptor Splice Variant 7 in Castrate-Resistant Prostate Cancer. Mol. Cell *72*, 341–354.e6.

15. Errichelli, L., Dini Modigliani, S., Laneve, P., Colantoni, A., Legnini, I., Capauto, D., Rosa, A., De Santis, R., Scarfò, R., Peruzzi, G., et al. (2017). FUS affects circular RNA expression in murine embryonic stem cell-derived motor neurons. Nat. Commun. *8*, 14741.

16. Conn, S.J., Pillman, K.A., Toubia, J., Conn, V.M., Salmanidis, M., Phillips, C.A., Roslan, S., Schreiber, A.W., Gregory, P.A., and Goodall, G.J. (2015). The RNA binding protein quaking regulates formation of circRNAs. Cell *160*, 1125–1134.

17. Fei, T., Chen, Y., Xiao, T., Li, W., Cato, L., Zhang, P., Cotter, M.B., Bowden, M., Lis, R.T., Zhao, S.G., et al. (2017). Genome-wide CRISPR screen identifies HNRNPL as a prostate cancer dependency regulating RNA splicing. Proc. Natl. Acad. Sci. USA *114*, E5207–E5215.

18. Zhou, X., Li, Q., He, J., Zhong, L., Shu, F., Xing, R., Lv, D., Lei, B., Wan, B., Yang, Y., et al. (2017). HnRNP-L promotes prostate cancer progression by enhancing cell cycling and inhibiting apoptosis. Oncotarget *8*, 19342–19353.

19. Peeters, J.G.C., Picavet, L.W., Coenen, S.G.J.M., Mauthe, M., Vervoort, S.J., Mocholi, E., de Heus, C., Klumperman, J., Vastert, S.J., Reggiori, F., et al. (2019). Transcriptional and epigenetic profiling of nutrient-deprived cells to identify novel regulators of autophagy. Autophagy *15*, 98–112.

20. Yang, Z., Qu, C.B., Zhang, Y., Zhang, W.F., Wang, D.D., Gao, C.C., Ma, L., Chen, J.S., Liu, K.L., Zheng, B., et al. (2019). Dysregulation of p53-RBM25-mediated circAMOTL1L biogenesis contributes to prostate cancer progression through the circAMOTL1L-miR-193a-5p-Pcdha pathway. Oncogene *38*, 2516–2532.

21. Jin, C., Zhao, W., Zhang, Z., and Liu, W. (2019). Silencing circular RNA circZNF609 restrains growth, migration and invasion by up-regulating microRNA-186-5p in prostate cancer. Artif. Cells Nanomed. Biotechnol. *47*, 3350–3358.

22. Hanniford, D., Ulloa-Morales, A., Karz, A., Berzoti-Coelho, M.G., Moubarak, R.S., Sánchez-Sendra, B., Kloetgen, A., Davalos, V., Imig, J., Wu, P., et al. (2020). Epigenetic Silencing of CDR1as Drives IGF2BP3-Mediated Melanoma Invasion and Metastasis. Cancer Cell *37*, 55–70.e15.

23. Chen, X., Chen, R.X., Wei, W.S., Li, Y.H., Feng, Z.H., Tan, L., Chen, J.W., Yuan, G.J., Chen, S.L., Guo, S.J., et al. (2018). PRMT5 Circular RNA Promotes Metastasis of Urothelial Carcinoma of the Bladder through Sponging miR-30c to Induce Epithelial-Mesenchymal Transition. Clin. Cancer Res. *24*, 6319–6330.

24. Yu, J., Xu, Q.G., Wang, Z.G., Yang, Y., Zhang, L., Ma, J.Z., Sun, S.H., Yang, F., and Zhou, W.P. (2018). Circular RNA cSMARCA5 inhibits growth and metastasis in hepatocellular carcinoma. J. Hepatol. *68*, 1214–1227.

25. Chen, J., Chen, T., Zhu, Y., Li, Y., Zhang, Y., Wang, Y., Li, X., Xie, X., Wang, J., Huang, M., et al. (2019). circPTN sponges miR-145-5p/miR-330-5p to promote proliferation and stemness in glioma. J. Exp. Clin. Cancer Res. *38*, 398.

26. Li, Q.H., Liu, Y., Chen, S., Zong, Z.H., Du, Y.P., Sheng, X.J., and Zhao, Y. (2019). circ-CSPP1 promotes proliferation, invasion and migration of ovarian cancer cells by acting as a miR-1236-3p sponge. Biomed. Pharmacother *114*, 108832.

27. Wang, Q., Shi, L., Shi, K., Yuan, B., Cao, G., Kong, C., Fu, J., Man, Z., Li, X., Zhang, X., et al. (2020). CircCSPP1 Functions as a ceRNA to Promote Colorectal Carcinoma Cell EMT and Liver Metastasis by Upregulating COL1A1. Front. Oncol. *10*, 850.

28. Shearer, R.F., Frikstad, K.M., McKenna, J., McCloy, R.A., Deng, N., Burgess, A., Stokke, T., Patzke, S., and Saunders, D.N. (2018). The E3 ubiquitin ligase UBR5 regulates centriolar satellite stability and primary cilia. Mol. Biol. Cell *29*, 1542–1554.

29. Hauge, H., Patzke, S., and Aasheim, H.C. (2007). Characterization of the FAM110 gene family. Genomics *90*, 14–27.

30. Sternemalm, J., Geimer, S., Frikstad, K.A., Schink, K.O., Stokke, T., and Patzke, S. (2015). CSPP-L Associates with the Desmosome of Polarized Epithelial Cells and Is Required for Normal Spheroid Formation. PLoS ONE *10*, e0134789.

31. Wang, S., Wu, J., Ren, J., Vlantis, A.C., Li, M.Y., Liu, S.Y.W., Ng, E.K.W., Chan, A.B.W., Luo, D.C., Liu, Z., et al. (2018). MicroRNA-125b Interacts with Foxp3 to Induce Autophagy in Thyroid Cancer. Mol. Ther. *26*, 2295–2303.

32. Li, P., He, J., Yang, Z., Ge, S., Zhang, H., Zhong, Q., and Fan, X. (2020). *ZNNT1* long noncoding RNA induces autophagy to inhibit tumorigenesis of uveal melanoma by regulating key autophagy gene expression. Autophagy *16*, 1186–1199.

OMTN, Volume *26*

Supplemental information

HnRNP-L-regulated circCSPP1/miR-520h/*EGR1* axis modulates autophagy and promotes progression in prostate cancer

Jianming Lu, Chuanfan Zhong, Junqi Luo, Fangpeng Shu, Daojun Lv, Zezhen Liu, Xiao Tan, Shuo Wang, Kaihui Wu, Taowei Yang, Weibo Zhong, Bin Wang, Yanfei Chen, Yuehan Li, Zhenyu Jia, Yaguang Zou, Weide Zhong, and Xiangming Mao

Supplementary Table 1 Sequence of primers, small RNAs and probes used in the study.

Primers		
Gene	**Forward (5'-3')**	**Reverse (5'-3')**
HnRNP-L	TTGTGGCCCTGTCCAGAGAATT	GTTTGTGTAGTCCCAAGTATCCTG
convergent-circCSPP1	TGTGAAAATTCAGAGGGTCCTA	TTGGAAATGCCCACTTCTTC
divergent-circCSPP1	TGAAGATTTGCGCAGTGGAC	GAGCATCCCTGCAAAAGGAC
convergent-GAPDH	GTCAGTGGTGGACCTGACCT	TGACAAAGTGGTCGTTGAGG
divergent-GAPDH	CACACTGAATCTCCCCTCCT	GTCCACCACCCTGTTGCT
CSPP1	TGAAGATTTGCGCAGTGGAC	GAGCATCCCTGCAAAAGGAC
GAPDH	ACAGTCAGCCGCATCTTCTT	GACAAGCTTCCCGTTCTCAG
EGR1	CACCTGACCGCAGAGTCTTTT	GGCCAGTATAGGTGATGGGG
β-actin	AGCGAGCATCCCCCAAAGTT	GGGCACGAAGGCTCATCATT
S1-pre-circCSPP1	TGGGCGACAGAGTGA	GAGTGGGACTTGGAAAC
S2-pre-circCSPP1	TCCCTCCCTTTCTTCT	ACAACTCAGAATAGAACCC
S3-pre-circCSPP1	TGTAGAGGAGCACTATTTCC	GAGTGGGACTTGGAAAC
S4-pre-circCSPP1	GTAGCTCTTTGCCTTCT	GTACAGGAGAAGGGACAT
S5-pre-circCSPP1	GTGCGGCATTACCAG	TGATCGCCATTCTAACT

siRNA/miRNA mimics/ inhibitors Sequence		
	**sense 5'-3'**	**antisense 5'-3'**
si-HnRNP-L	GCUUGGAUCAAUCUAAGAUTT	AUCUUAGAUUGAUCCAAGCTT
circCSPP1-sh1	AUGGUGUCUCCCAGUGCUCTT	GAGCACUGGGACACACCAUTT
circCSPP1-sh2	GUCUCCCAGUGCUCCAGACTT	GUCUGGAGCACUGGGAGACTT
hsa_miR-520h mimics	ACAAAGUGCUUCCCUUUAGAGU	UCUAAAGGGAAGCACUUUGUUU
hsa_miR-520h inhibitors	ACUCUAAAGGGAAGCACUUUGU	

FISH probes targeting circCSPP1/miR-520h/*EGR1*		
	**Sequence 5'-3'**	**modification**
circCSPP1	TGTTTCATTGTCTGGAGCACTGGGAGACACCATTTCACCA	CY3/FAM
hsa_miR-520h	ACTCTAAAGGGAAGCACTTTGT	CY3
EGR1	AAAAGCGGCCAGTATAGGTGAT	FAM

Pulldown probes targeting circCSPP1/miR-520h		
	**Sequence 5'-3'**	**modification**
circCSPP1	UUCAUUGUCUGGAGCACUGGGAGACACCAUUUCACC	5’ biotin
Oligo probes	UUCUCCGAACGUGUCACGUTT	5’ biotin
hsa_miR-520h_sense	ACAAAGUGCUUCCCUUUAGAGU	3’ biotin
hsa_miR-520h_antisense	ACUCUAAAGGGAAGCACUUUGU	-

Supplementary Table 2 Clinical characteristics of the prostate cancer patients.

Parameter			
Age at diagnosis (mean ± SD)		72 ± 3.53	
Clinical stage, n(%)	T1	1	10%
	T2	6	60%
	T3	3	30%
Gleason score, n(%)	≤6	3	30%
	=7	3	30%
	≥8	4	40%
PSA at diagnosis (ng/ml), n(%)	0-3.9	1	10%
	4-9.9	3	30%
	≥10	6	60%

Supplementary Table 3 Clinical characteristics of the benign prostate hyperplasia.

Parameter			
Age at diagnosis (mean ± SD)		70.7 ± 3.92	
Pathological diagnosis, n (%)	Benign	10	100%
	Cancer	0	0%
PSA at diagnosis (ng/ml), n (%)	0-3.9	10	100%
	4-9.9	0	0%
	≥10	0	0%

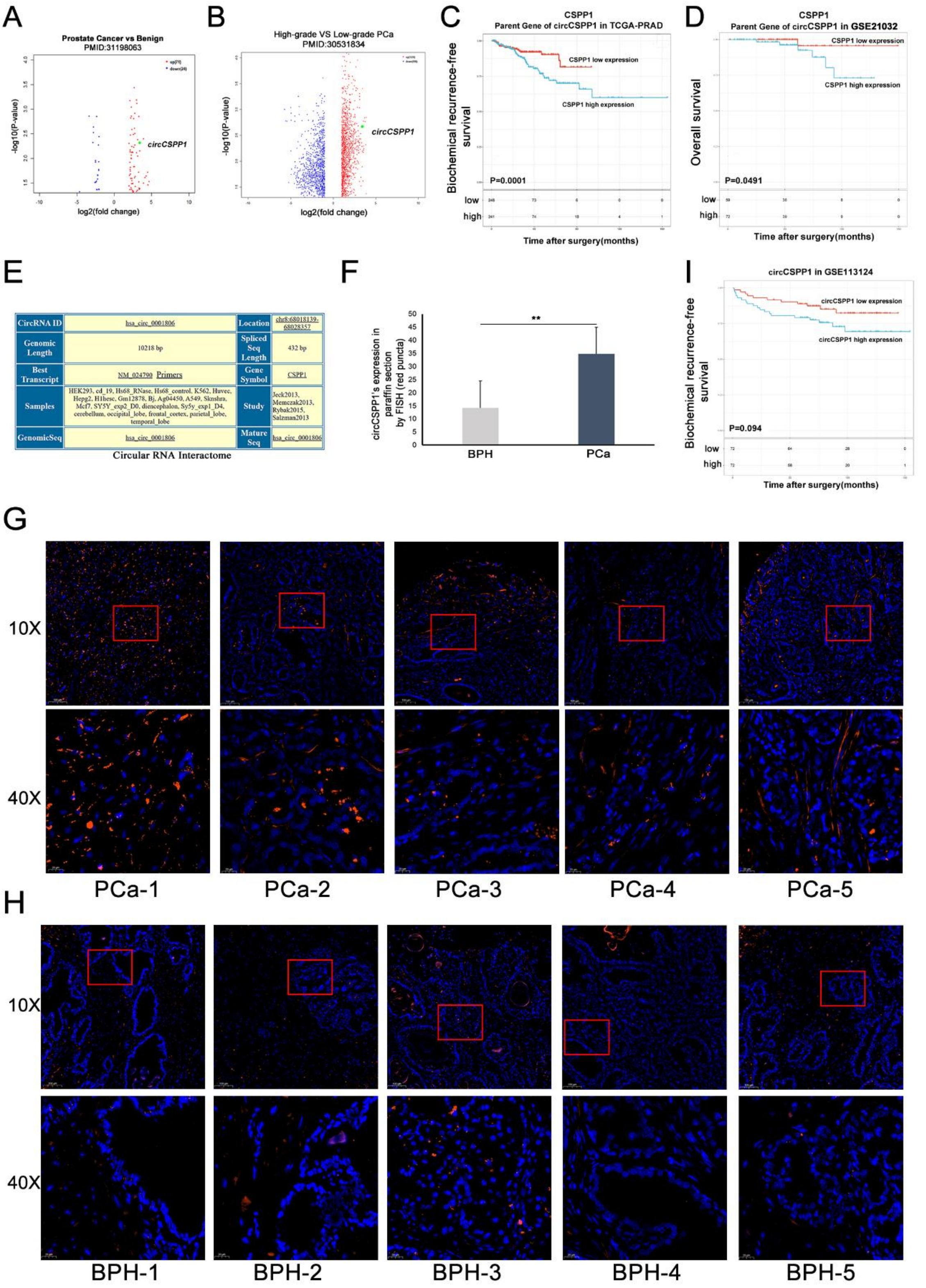

Supplementary Figure 1 circCSPP1 is upregulated in PCa. (A) The scatter diagram shows the most differentially expressed circRNAs in PCa tissues and benign tissues. (B) Dysregulated circRNAs expression were shown in 5 pairs of High-grade and low-grade PCa samples. (C) Biochemical recurrence-free survival time about parent gene of circCSPP1 analysis in TCGA database (D) Overall survival (OS) time data for prostate

cancer patients from the Kaplan-Meier Plotter analysis. (E) Annotation of circCSPP1 from Circular RNA Interactome. (F) Expression of circCSPP1 in clinical samples. (G) circCSPP1 is detected by FISH in PCa paraffin section (DAPI, blue puncta; circCSPP1, red puncta). (H) circCSPP1 is detected by FISH in BPH paraffin section (DAPI, blue puncta; circCSPP1, red puncta). (I) KM plot indicated the high circCSPP1 expression is correlated with faster BCR in patients with PCa though the log-rank p value = 0.094. Data are shown as means ± SD, Student's t test, two-tailed, **P<0.01.

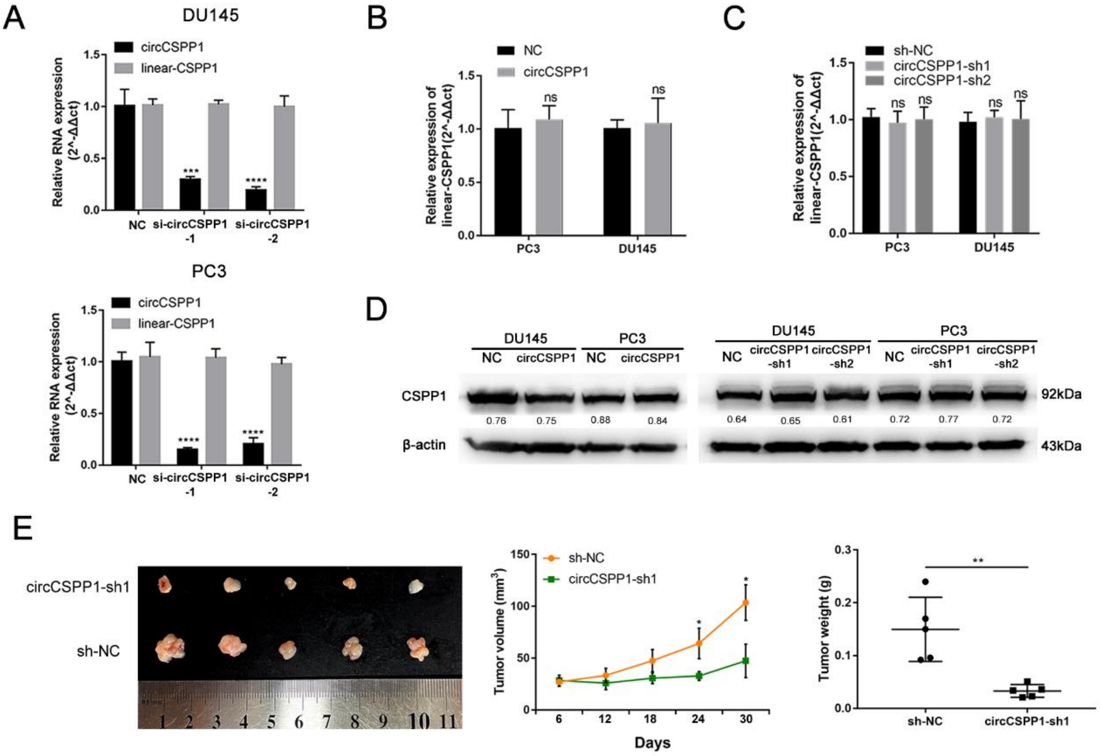

Supplementary Figure 2 circCSPP1 modulates PCa proliferation positively *in vivo* without interfering the expression of linear-*CSPP1*. (A) qRT-PCR validation for the transfection efficiency of siRNA targeting circCSPP1 and its influence on the expression of linear-CSPP1. (B, C) qRT-PCR validation for the transfection efficiency of lentivirus expressing circCSPP1 and sh-circCSPP1 and their impacts on the expression of linear-*CSPP1*. (D) Overexpression or knockdown of circCSPP1 barely affected the protein expression of CSPP1. (E) Image of subcutaneous tumors derived from PC3 cell transfected with vector or circCSPP1-sh1 in the xenograft model. Tumor volumes were measured every 6 days up to 30 days and the final tumor weight was calculated. Data are shown as means ± SD, Student's t test, two-tailed, ****P<0.0001, ***P<0.001, **P<0.01,*P<0.05, ns, not significant.

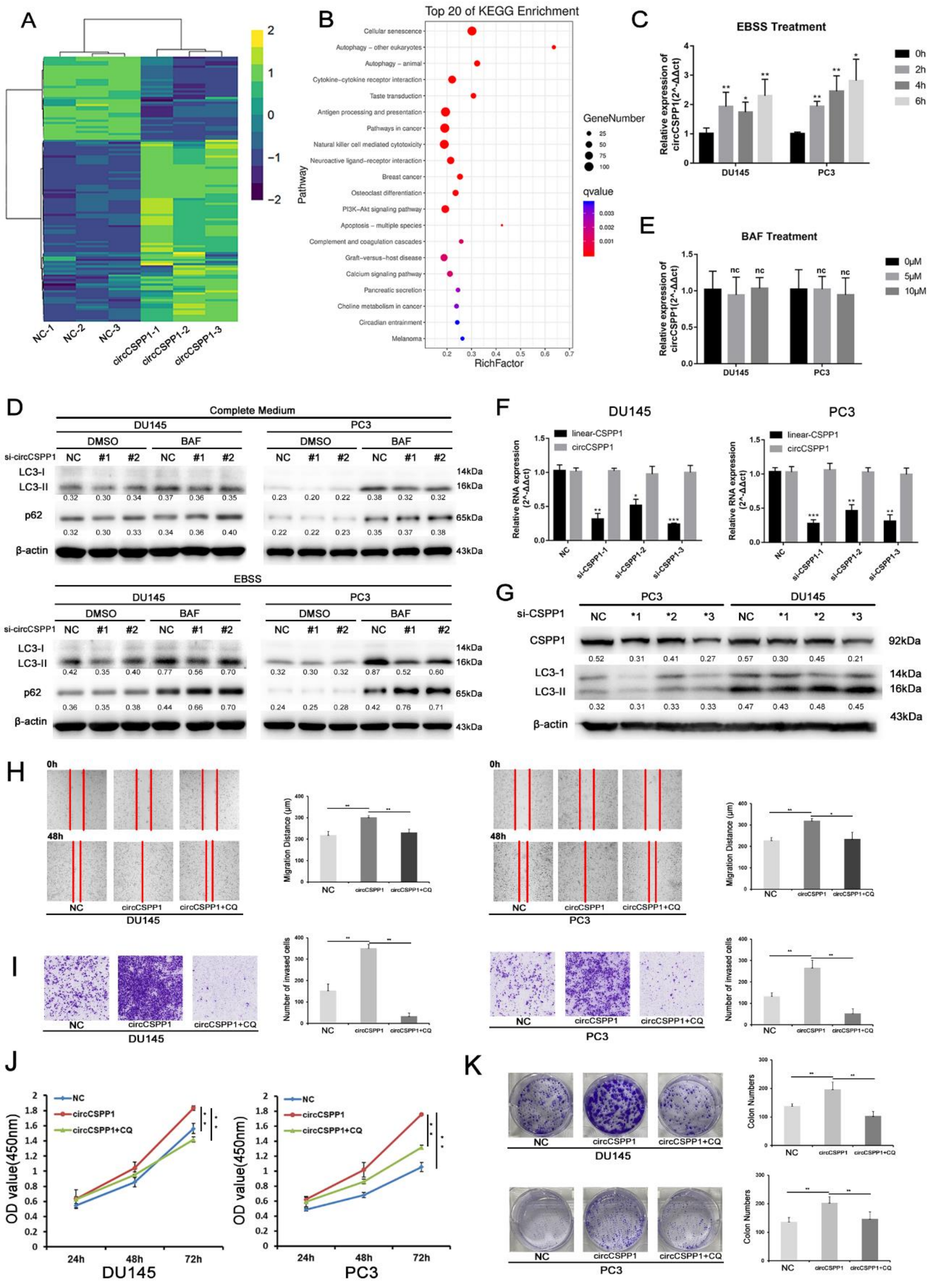

Supplementary Figure 3 circCSPP1 promotes PCa progression via activating autophagy. (A) Heatmap representing unsupervised hierarchical clustering of genes regulated by circCSPP1 based on next-generation sequencing (GSE158975) analysis. (B) KEEG analysis displays the enrichment pathways in circCSPP1 overexpressed cell lines.

(C) Increments of circCSPP1 over time by autophagy induction with EBSS. (D) Western blotting detected the conversion of LC3-II and P62 in complete medium or EBSS condition under DMSO/BAF treatment after si-circCSPP1 transfection. (E) Expression of circCSPP1 remains unchanged under treatment with BAF in different concentration. (F) qRT-PCR validation for the transfection efficiency of siRNA targeting linear-*CSPP1* and its impact on the expression of circCSPP1. (G) Knockdown of linear-*CSPP1* significantly decreased the protein level of CSPP1 but exhibited no effect on LC3-II and P62 expression. (H, I) The migration and invasion capabilities of DU145 and PC3 transfected with circCSPP1 were determined by the wound healing and transwell assays with or without CQ. (J, K) The proliferative ability of DU145 and PC3 transfected with circCSPP1 was determined through the colony formation and CCK-8 assays with or without CQ. Data are shown as means ± SD, Student's t test, two-tailed, ***$P<0.001$, **$P<0.01$, *$P<0.05$, ns, not significant.

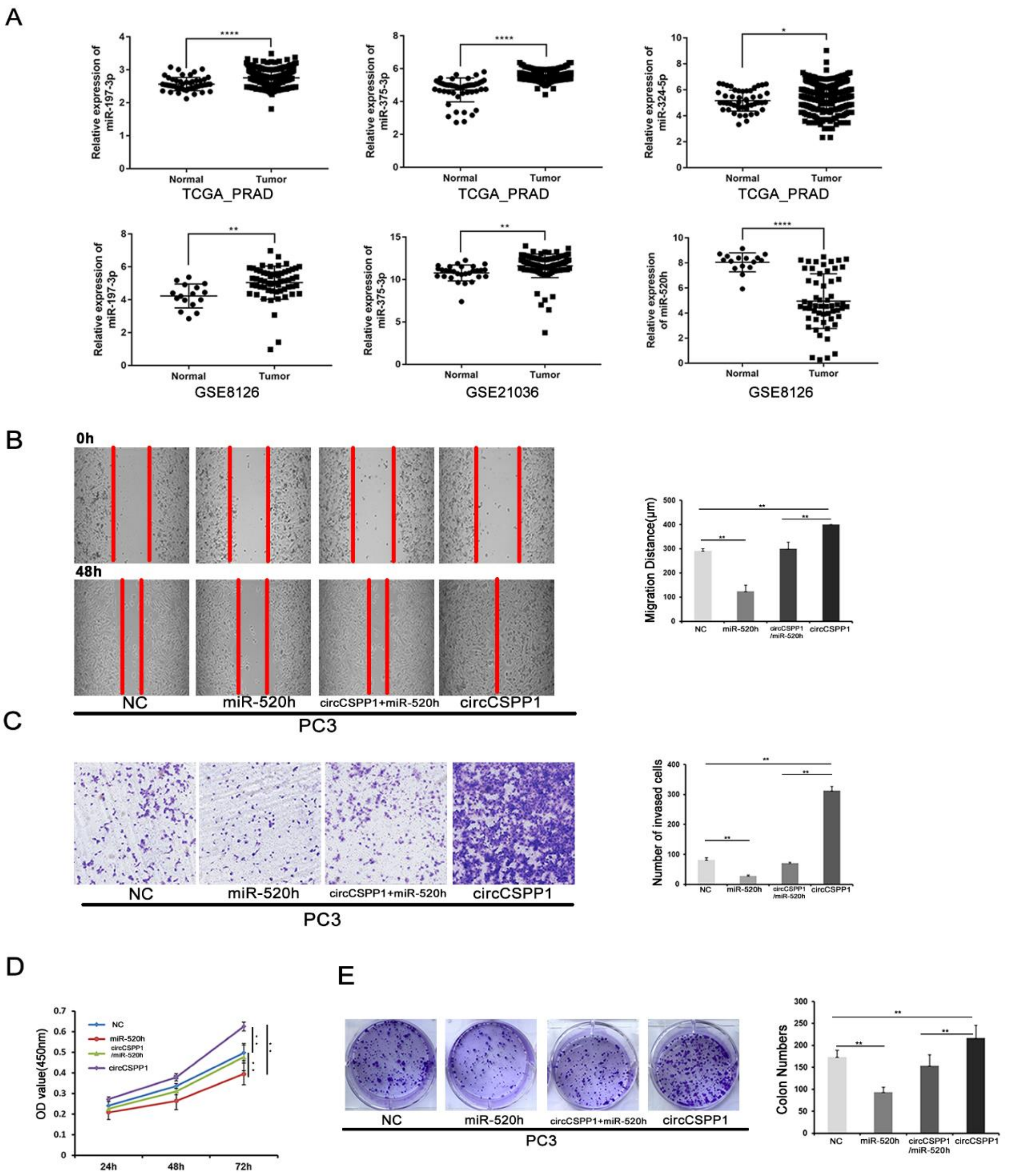

Supplementary Figure 4 circCSPP1 promotes PCa progression through sponging off miR-520h. (A) Differentially expression of putative miRNAs in TCGA and GEO databases, and miR-520h was downregulated in PCa while miR-432-5p was unknown. (B, C) The migration and invasion capabilities of DU145 and PC3 transfected with circCSPP1 and/or miR-520h mimics were determined with the wound healing and transwell assays. (D, E) The proliferative ability of DU145 and PC3 transfected with circCSPP1 and/or miR-520h mimics was determined through the colony formation and CCK-8 assays. Data are shown as means ± SD, Student's t test, two-tailed, ****P<0.0001, **P<0.01, *P<0.05, ns, not significant.

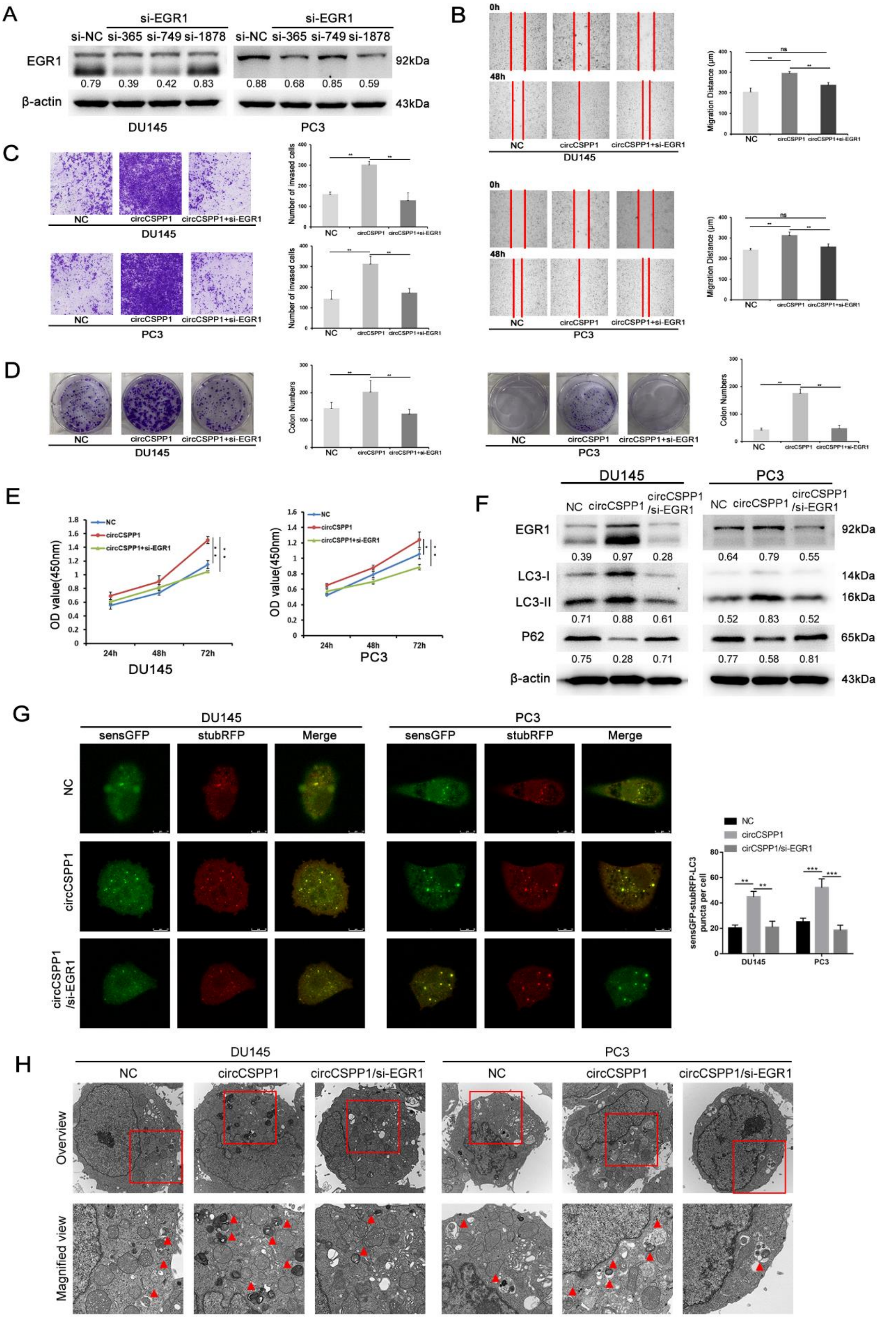

Supplementary Figure 5 Knockdown of *EGR1* attenuated circCSPP1 induced progression and autophagy in PCa *in vitro*. (A) Western blot validation for the

transfection efficiency of siRNA targeting *EGR1*. (B, C) The migration and invasion capabilities of DU145 and PC3 transfected with vector or circCSPP1, or co-transfected with circCSPP1 and si-*EGR1* were determined with the wound healing and transwell assays. (D, E) The proliferative ability of DU145 and PC3 transfected with vector or circCSPP1, or co-transfected with circCSPP1 and si-*EGR1* was determined through the colony formation and CCK-8 assays. (F) Western blotting verified knockdown of *EGR1* attenuated the circCSPP1 induced increment of the conversion of LC3-I/LC3-II and P62 degradation. (C) The accumulation of LC3-II puncta (yellow, green and red overlap) was detected in DU145 and PC3 after transfection of circCSPP1 or co-transfection of circCSPP1 and si-*EGR1*. (D) Autophagosomes (arrow) were observed by transmission electron microscopy (TEM) in DU145 and PC3 after transfection of circCSPP1 or co-transfection of circCSPP1 and si-*EGR1*. Data are shown as means ± SD, Student's t test, two-tailed, ***P<0.001, **P<0.01, *P<0.05, ns, not significant.

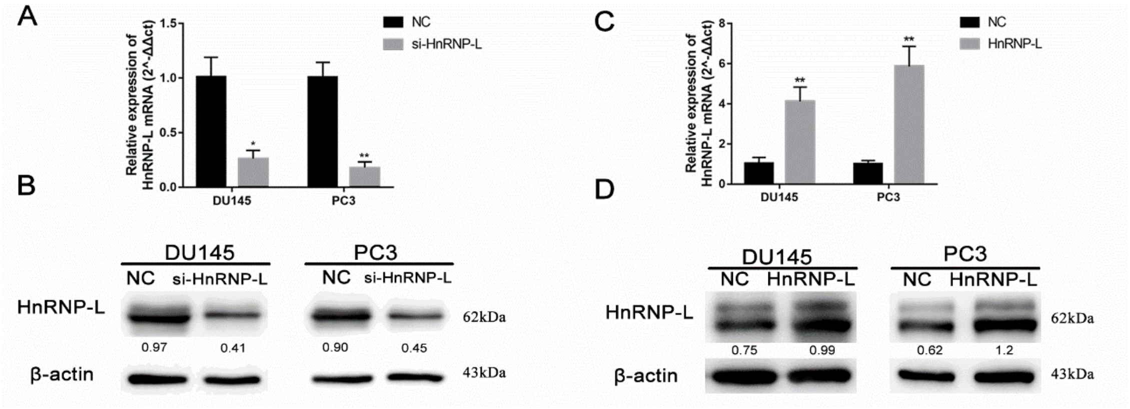

Supplementary Figure 6 Transfection efficiency of si-HnRNP-L and HnRNP-L overexpression in DU145 and PC3 cell lines. (A, B) qRT-PCR and Western blot validation for the transfection efficiency of siRNA targeting HnRNP-L. (C, D) qRT-PCR and Western blot validation for the transfection efficiency of lentivirus expressing HnRNP-L. Data are shown as means ± SD, Student's t test, two-tailed, **P<0.01, *P<0.05.

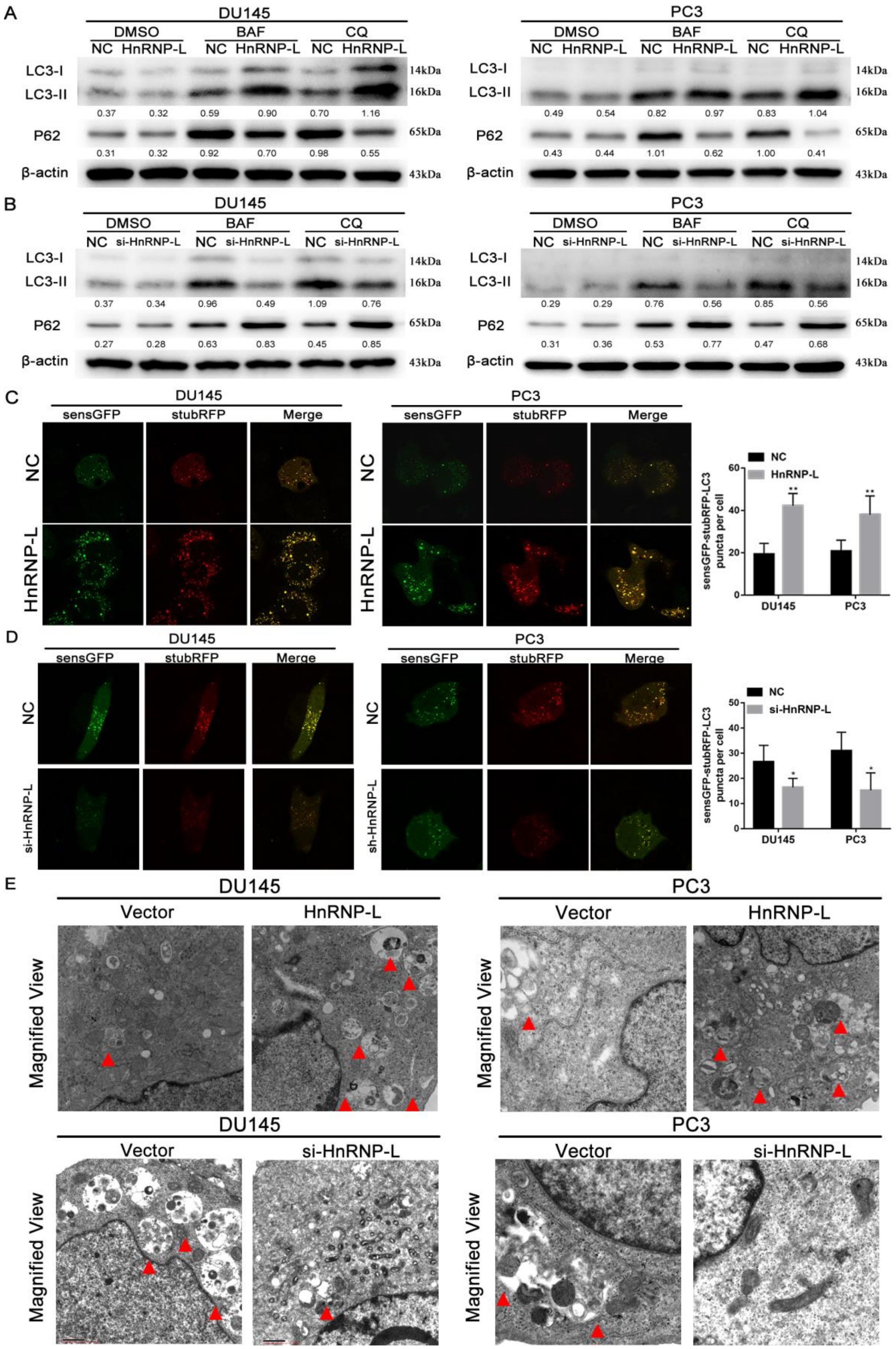

Supplementary Figure 7 Overexpression or knockdown of HnRNP-L augmented or impeded autophagy in DU145 and PC3 cell lines. (A, B) Western blotting verified

overexpression or knockdown of HnRNP-L promoted or inhibited the conversion of LC3-I/LC3-II and P62 degradation. (C) The accumulation of LC3-II puncta (yellow, green and red overlap) was detected in DU145 and PC3 after overexpression or knockdown of HnRNP-L. (D) Autophagosomes (arrow) were observed by transmission electron microscopy (TEM) in DU145 and PC3 after overexpression or knockdown of HnRNP-L. Data are shown as means ± SD, Student's t test, two-tailed, $**P<0.01$, $*P<0.05$.
